# HIV-1 gp120 Mannoses Induce Immunosuppressive Responses from Dendritic Cells

**DOI:** 10.1371/journal.ppat.0030169

**Published:** 2007-11-02

**Authors:** Meimei Shan, Per Johan Klasse, Kaustuv Banerjee, Antu K Dey, Sai Prasad N Iyer, Robert Dionisio, Dustin Charles, Lila Campbell-Gardener, William C Olson, Rogier W Sanders, John P Moore

**Affiliations:** 1 Department of Microbiology and Immunology, Weill Medical College of Cornell University, New York, New York, United States of America; 2 Progenics Pharmaceuticals Incorporated, Tarrytown, New York, United States of America; 3 Laboratory of Experimental Virology, Department Medical Microbiology, Center of Infection and Immunity Amsterdam (CINIMA), Academic Medical Center of the University of Amsterdam, Amsterdam, The Netherlands; The Scripps Research Institute, United States of America

## Abstract

The human immunodeficiency virus type 1 (HIV-1) envelope glycoprotein gp120 is a vaccine immunogen that can signal via several cell surface receptors. To investigate whether receptor biology could influence immune responses to gp120, we studied its interaction with human, monocyte-derived dendritic cells (MDDCs) in vitro. Gp120 from the HIV-1 strain JR-FL induced IL-10 expression in MDDCs from 62% of donors, via a mannose C-type lectin receptor(s) (MCLR). Gp120 from the strain LAI was also an IL-10 inducer, but gp120 from the strain KNH1144 was not. The mannose-binding protein cyanovirin-N, the 2G12 mAb to a mannose-dependent gp120 epitope, and MCLR-specific mAbs inhibited IL-10 expression, as did enzymatic removal of gp120 mannose moieties, whereas inhibitors of signaling via CD4, CCR5, or CXCR4 were ineffective. Gp120-stimulated IL-10 production correlated with DC-SIGN expression on the cells, and involved the ERK signaling pathway. Gp120-treated MDDCs also responded poorly to maturation stimuli by up-regulating activation markers inefficiently and stimulating allogeneic T cell proliferation only weakly. These adverse reactions to gp120 were MCLR-dependent but independent of IL-10 production. Since such mechanisms might suppress immune responses to Env-containing vaccines, demannosylation may be a way to improve the immunogenicity of gp120 or gp140 proteins.

## Author Summary

Dendritic cells (DCs) initiate immune responses to pathogens or vaccine antigens. The HIV-1 gp120 envelope glycoprotein is an antigen that is a focus of vaccine design strategies. We have studied how gp120 proteins interact with DCs in cell culture. Certain gp120s stimulate DCs from some, but not all, human donors to produce IL-10, a cytokine that is generally immunosuppressive. In addition, whether or not the DCs produce IL-10, their ability to mature properly when activated is impaired by gp120—the gp120-treated DCs have a reduced ability to stimulate T cell growth when the two cell types are cultured together. These various effects of gp120 are caused by its binding to cell surface receptors of the mannose C-type lectin receptor family, including (but probably not exclusively) one called DC-SIGN. Gp120 binds to these receptors via mannose residues that are present on some of the glycan structures that overlay much of its protein surface. Removing the mannoses by digesting gp120 with a suitable enzyme prevents IL-10 induction and impairment of DC maturation, as does the use of inhibitors of the binding of gp120 to DC-SIGN and similar receptors. This work could help with the design of better HIV-1 vaccines.

## Introduction

One approach to a vaccine against HIV-1 is the use of the viral envelope glycoproteins (Env) as immunogens to induce neutralizing antibodies (NAbs) [1–3]. Usually, the Env glycoproteins are presented as adjuvanted, soluble proteins after production in vitro as recombinant proteins, but they can also be expressed in vivo from delivery systems based on DNA or live recombinant viruses (e.g., poxvirus or adenovirus vectors) [4]. Different configurations of Env glycoproteins have been studied as vaccine antigens, initially the surface glycoprotein gp120; more recently, soluble oligomeric gp140 proteins based broadly on the native gp120-gp41 complex [1–3].

Irrespective of how HIV-1 Env glycoproteins have been presented and in whatever configuration, the induction of broadly active NAbs has proven problematic [1]. One generally accepted problem is the evolution of the native Env complex into a configuration that limits the exposure of the few neutralization sites that are present. The potential solution is to further understand the structure of the complex, then to engineer antigens that are better able to present relevant NAb epitopes to the immune system; attempts to do this are in progress in many laboratories worldwide [1]. Here, however, we focus on what we consider to be another factor hindering NAb induction: the limited immunogenicity of HIV-1 Env proteins in general.

Although antibody responses to HIV-1 Env can clearly be induced in infected or vaccinated humans and animals, these proteins are not particularly immunogenic. Thus, gp120 or gp140 proteins are typically administered at 100–500 μg per dose, and the binding antibody titers raised against them can be highly variable; some humans and animals respond fairly well, others only poorly [5–9]. Anti-Env antibody titers also decay rather rapidly (half-lives are typically in the range 30–50 d) and frequent boosting is required to maintain them. Few directly comparative studies have ever been performed, but the limited information available supports the contention that Env is an unusually problematic immunogen compared to most other vaccine antigens [10] (S. Plotkin and B. Graham, personal communication).

The immune responses to HIV-1 Env vaccine antigens are T_H_2-polarized to an extent that is unusual even for a soluble protein [11,12]. The same T_H_2 bias can also be observed during HIV-1 infection, although this is a much more complex and controversial situation [13–15]. The nature of the immune response to gp120 may be attributable to the fundamental properties of this unusual protein. One feature that distinguishes gp120 from many other vaccine immunogens is its biological activity; gp120 can bind to several cell surface receptors: CD4, CCR5, CXCR4, and several mannose C-type lectin receptors (MCLR) including but not limited to DC-SIGN [2]. In vitro, one consequence of gp120 binding to such receptors is the transduction of intracellular signals that can have many different, but generally adverse, effects on the various target cells. Although the gp120 concentrations used to elicit such signals (μg/ml range) are usually grossly in excess of what could be present in serum during HIV-1 infection [16], they are compatible with what is used for immunization (several hundred μg of protein delivered in a few ml into a localized tissue site) [5–9]. We therefore considered it possible that gp120 immunization could trigger signals affecting how an immune response develops. For example, one cellular response to gp120 in vitro is the induction of IL-10, an anti-inflammatory cytokine [17–24]. Here, we have studied what happens when gp120 interacts with human monocyte-derived dendritic cells (MDDCs) in vitro. We show that a consequence of JR-FL gp120 binding to these cells from ∼50% of donors is the induction of IL-10. Moreover, gp120-treated MDDCs impair the proliferation of co-cultured CD4^+^ T cells and reduce their expression of IL-12. These responses are also a consequence of the mannose-dependent interaction of gp120 with an MCLR, although they are not obligatorily linked to IL-10 expression. The various outcomes of gp120-MCLR interactions are prevented by enzymatic removal of gp120 mannoses, a method that may improve the immunogenicity of HIV-1 Env proteins and some other vaccine-relevant immunogens.

## Results

### HIV-1 gp120 Induces MDDCs to Produce IL-10

We investigated how gp120 affected MDDC maturation and cytokine secretion, and MDDC-T cell interactions in view of the key role dendritic cells (DCs) play in antigen capture, processing, and presentation. The preparation and properties of the MDDCs are described under Supporting Information (Figure S1). We were particularly interested to ascertain whether gp120 induced IL-10 expression in MDDCs, in view of the immunosuppressive effects of IL-10 and its role in T_H_2-polarization of responses to gp120 in immunized mice [11], and the induction of IL-10 by gp120 in human monocyte/macrophages in vitro [17,18,20,22,24]. We therefore used MDDCs that were immature at the start of the experiment (iMDDCs), to enable us to monitor the subsequent maturation process. However, in some studies, we investigated the effects of gp120 on MDDC that were simultaneously induced to mature by other stimuli, notably lipopolysaccharide (LPS).

**Figure 1:**
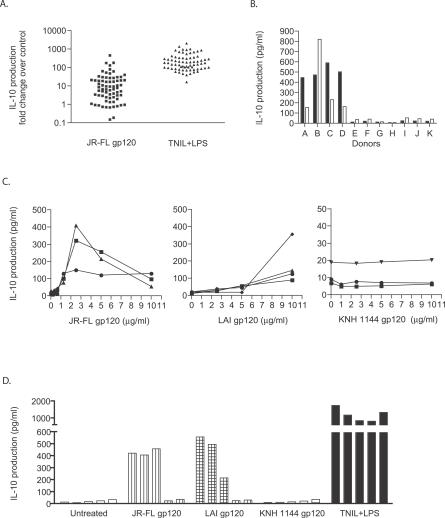
HIV-1 gp120 Induces IL-10 Secretion from MDDCs in a Donor- and Concentration-Dependent Manner (A) MDDCs from different human donors were cultured in GM-CSF + IL-4 for 6 d and then incubated for 24 h (*n* = 71 donors) with or without JR-FL gp120 (3 μg/ml) before measurement of IL-10 production by ELISA. The fold-increases in IL-10 production after gp120 treatment compared with untreated cells are depicted on the *y*-axis. Another set of MDDC from each donor was stimulated for 24 h with TNIL + LPS (±CD40L), instead of gp120. The corresponding fold-increase in IL-10 secretion compared with untreated cells is also plotted. (B) MDDCs from 11 pedigreed donors were cultured and stimulated with gp120 or TNIL + LPS, as in (A), twice within a 3-mo interval. IL-10 production was measured 24 h after stimulation. Black bars represent the first assay on each donor, white bars the second. (C) The secretion of IL-10 from iMDDCs after 24 h is depicted as a function of the gp120 concentration. The data points show mean values ± SD (the error bars lie within the symbols) of duplicate ELISA determinations. Each curve represents data derived from a single donor, the same symbol representing the same donor in each panel. Left: Three different donors, JR-FL gp120. Middle: Four different donors, LAI gp120. Right: Three different donors, KNH1144 gp120 (the square symbol is overlaid by the circle symbol). (D) iMDDCs from five donors were treated with JR-FL gp120 (3 μg/ml), LAI gp120 (10 μg/ml), KNH1144 gp120 (10 μg/ml), or TNIL + LPS before measurement of IL-10 production at 24 h.

iMDDCs from a day-6 culture were washed thoroughly to prevent further stimulation with IL-4 and GM-CSF, then incubated for two further days with or without CHO-cell expressed, JR-FL (R5) gp120 (the 3 μg/ml; 25 nM concentration was based on titrations in pilot studies; see below). In the absence of any stimulus, the cells produced little IL-10 (mean 11 ± 2.5 pg/ml at 24 h, *n* = 71 and 28 ± 3.8 pg/ml at 48 h, *n* = 52) and no detectable IL-12p70 over a 48-h period starting on day 6. The addition of JR-FL gp120 triggered significant IL-10 secretion from MDDCs from a subset of the 71 blood donors (Figure 1A). Thus, 24 h later, IL-10 production was increased by >5-fold in MDDCs from 62% (44/71) donors, with the median increase being 8.5-fold (median control value: 7.5 pg/ml; median + gp120, 64 pg/ml). Similar responses were observed at 48 h (median control value: 17 pg/ml; + gp120, 98 pg/ml). The IL-10 increases triggered by gp120 were significant at both 24 h and 48 h (Mann-Whitney U test, one tail, *p* < 0.0001). However, MDDCs from 38% of the donors did not respond to gp120 (IL-10 increases of <5-fold).

Although a subset was unresponsive to gp120, day-6 iMDDCs from every donor reacted to the classic TNIL + LPS (+CD40L when IL-12p70 was analyzed) maturation stimulus by producing high levels of both IL-10 (mean 1,639 ± 665 pg/ml, *n* = 71) and IL-12p70 (mean 235 ± 56 pg/ml, *n* = 12) over a 48-h period (Figure 1A and unpublished data). The median fold-increase in IL-10 production in response to TNIL + LPS after 24 h was 149-fold, 17.5 times greater than the median response to gp120. The IL-10 responses to TNIL + LPS and to gp120 did not correlate (at 24 h, *n* = 71, *r^2^* = 0.0007 and at 48 h, *n* = 12, *r^2^* = 0.006, respectively). The time courses of the IL-10 responses to JR-FL gp120, at both the mRNA and protein levels, and to TNIL + LPS at the mRNA level, are shown as Figure S2.

The donor-dependent variation in the IL-10 response to gp120 could be explained by genetic or epigenetic factors. As a first step to determining which applied, we performed experiments on MDDCs from 11 repeat donors, at two time points, 1–3 months apart. An IL-10 response to gp120 was observed in MDDCs from four of the 11 donors at both time points, whereas there was no response at either time point from cells of the other seven donors (Figure 1B). The consistency of the response pattern is more suggestive of a genetic or a constant epigenetic determinant than of a variable epigenetic factor such as, for example, an inter-current infection.

IL-10 secretion by MDDCs from responsive donors was dependent on the concentration and the identity of the gp120 protein used (Figure 1C). The optimal response to JR-FL gp120 occurred at 3 μg/ml, whereas the dose-response curve for LAI gp120 was slightly different, IL-10 secretion being greatest at 10 μg/ml, the highest concentration tested. However, when MDDCs from the same donors were exposed to gp120 from the subtype A virus KNH1144, there was no IL-10 response (Figure 1C). Furthermore, when MDDCs from five donors were tested comparatively, the same three that responded to JR-FL gp120 also did so to LAI gp120, and to similar extents, whereas MDDCs from all five donors responded to TNIL + LPS by producing high levels of IL-10 (Figure 1D). Hence both viral and host genetics may influence whether MDDCs produce IL-10 after exposure to gp120. We determined the viability of iMDDCs and mature MDDCs (mMDDCs) exposed for 48 h to the three different gp120s by staining with 7 amino-actinomycin D. Spontaneous cell death in cultures from different donors varied from 2%–3% in iMDDCs and 3%–12% in mMDDCs. The additional death of iMDDCS or mMDDCS measured in the presence of up to 10 μg/ml of JR-FL gp120, with or without demannosylation (see below), was <7%; for LAI gp120, it was <5% at up to 20 μg/ml; for KNH1144 gp120 it was <5% at up to 10 μg/ml.

The JR-FL, LAI, and KNH1144 proteins used in Figure 1C were all manufactured under good manufacturing process conditions and were essentially LPS-free. We also tested several additional gp120 proteins of different genotypes and expressed in different cell types (including insect cells) that we obtained from commercial sources and academic collaborators. In general, the degree of LPS contamination in these preparations was too high for the results to be interpretable, since LPS is itself a highly efficient inducer of IL-10 from MDDCs (Figure 1).

### HIV-1 gp120 Stimulates IL-10 Production by MDDCs through a Mannose-Dependent Interaction

**Figure 2:**
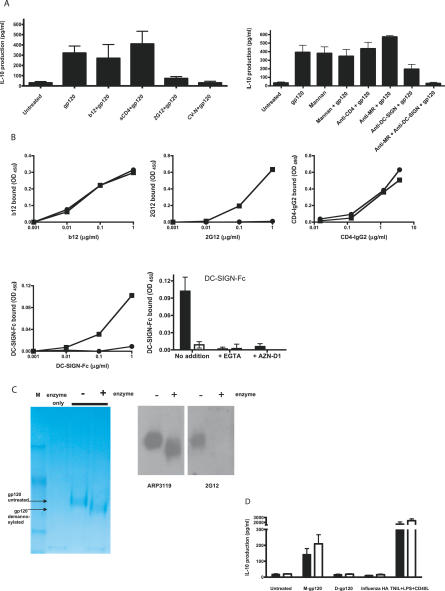
The Induction of IL-10 Secretion by gp120 Is Mannose-Dependent (A) The bars represent IL-10 production from MDDCs on day 6 after 24 h (black bars) of treatment with JR-FL gp120 (3 μg/ml). The reagents listed on the horizontal axis were incubated with gp120 or iMDDCs for 1 h prior to addition of gp120 to the cells (see Materials and Methods for the inhibitor concentrations tested). The bars represent the mean value ± SEM for data derived from five different, gp120-responsive donors. The left and right panels show data derived from different experiments. The various reagents were also tested in the absence of gp120 and found not to stimulate IL-10 production (<25 pg/ml, unpublished data), with the exception of mannan (see right panel). (B) The reactivities of mAbs b12 and 2G12, the CD4-IgG2 protein, or DC-SIGN-Fc with mock-treated JR-FL M-gp120 (squares) and α-(1,2,3,6) mannosidase-treated JR-FL D-gp120 (circles) were compared using ELISAs. In the fifth panel, the binding of DC-SIGN-Fc to JR-FL gp120 was inhibited by the Ca^2+^-chelator EGTA and the anti-DC-SIGN mAb, AZN-D1 (M-gp120 black bar; D-gp120 white bar). (C) Left panel: A reducing SDS-PAGE gel shows the reduction in JR-FL gp120 m.wt. caused by treatment with α-(1,2,3,6) mannosidase. Right panel: western blotting with anti-gp120 serum ARP3119 confirms the m.wt reduction, and blotting with mAb 2G12 shows that its mannose-dependent epitope has been removed from gp120. (M = m.wt markers; enzyme only = no gp120 present). (D) The experimental design was the same as in (A). The gp120 proteins (or influenza HA or TNIL + LPS + CD40L) tested are listed on the *x*-axis. The bars represent the mean values ± SEM for data derived from five different donors (black bars, IL-10 production after 24 h; white bars, after 48 h).

To determine which gp120 receptors on iMDDCs were responsible for activating IL-10 expression, we incubated either gp120 or the cells with ligands that should block known gp120-receptor interactions (Figures 2A, S3, and S4). Neither the b12 mAb to the CD4-binding site on gp120 nor sCD4 inhibited IL-10 production, implying that a gp120-CD4 interaction was not responsible. The small-molecule CCR5 antagonist AD101 was not inhibitory, ruling out signals transduced via gp120-CCR5 binding (Figure S4). The CXCR4 antagonist AMD3100 was inactive against IL-10 induction by gp120 from the X4 virus, LAI, so CXCR4 is also uninvolved (unpublished data). As expected, AMD3100 did not inhibit the IL-10 response to JR-FL gp120, or AD101 the response to LAI gp120 (Figure S4 and unpublished data). In contrast, when gp120 was pre-treated with either mAb 2G12 or CV-N, IL-10 induction was strongly inhibited (Figure 2A). Both 2G12 and CV-N bind to mannose moieties on gp120 N-linked glycans [25–27], implicating an interaction between gp120 and an MCLR(s) as the critical trigger for IL-10 induction. We also tested whether soluble mannans antagonized gp120-dependent IL-10 expression, but found that mannans themselves strongly activated an IL-10 response (Figure 2A). However, combining gp120 with mannans did not further elevate IL-10 levels, suggesting that both of these mannose-containing ligands bind to, and saturate, the same MCLR(s). To explore which MCLR(s) might be involved, we used mAbs specific to DC-SIGN and the mannose receptor (MR). The anti-DC-SIGN mAb AZN-D1 completely blocks the binding of mannosylated gp120 to DC-SIGN in an ELISA (Figure 2B). Two mAbs to DC-SIGN, including AZN-D1 and mAb Clone 19 to the MR, can each reduce the binding of gp120 to a subset of tonsillar B cells [28]. When the anti-DC-SIGN and anti-MR mAbs AZN-D1 and Clone 15–2 were each pre-incubated with iMDDCs, AZN-D1 partially (∼50%) reduced gp120-mediated IL-10 induction whereas Clone 15–2 was not inhibitory; adding the two mAbs together completely abolished the IL-10 response (Figure 2A). An anti-CD4 mAb was not inhibitory by itself at 24 h and did not affect the actions of the anti-MCLR mAbs when combined with them, although it did cause partial (45% ± SD 29%) inhibition at 48 h (Figure 2A and unpublished data). Other than mannan, the various mAbs and ligands described above did not induce IL-10 expression or block LPS-induced IL-10 expression (Figures 2A, S4, and unpublished data). The same concentrations (40 μg/ml) of different murine isotype control antibodies, alone and in combination, were also without effect (Figure S4 and unpublished data).

The above experiments imply that MCLRs, particularly but probably not only DC-SIGN, are the gp120 receptors that trigger the IL-10 response. If so, the high mannose residues on gp120 glycans are likely to be the cognate ligands. To prove this, the mannose moieties were removed from gp120 by enzymatic digestion with α-(1,2,3,6)-mannosidase [25]. Reducing SDS-PAGE gel analysis showed the demannosylated JR-FL gp120 (D-gp120) was slightly smaller than mock-treated gp120 (M-gp120; processed without the enzyme) and had lost its 2G12 epitope (Figure 2C; compare lanes marked + and −). The successful removal of mannose was verified by showing that D-gp120 failed to bind either 2G12 or DC-SIGN-Fc in ELISAs, in contrast to M-gp120 (Figure 2B). However, both mAb b12 and CD4-IgG2 bound efficiently to structures associated with the CD4-binding site on D-gp120 (Figure 2B), showing that JR-FL gp120 was efficiently demannosylated without impairing its overall conformation [25,27]. Hence we could now directly assess the role of the high mannose glycans in the IL-10 response.

M-gp120 induced substantial IL-10 production (150–300 pg/ml) from MDDCs from five different donors, whereas D-gp120 had no such effect. Influenza virus HA did not stimulate IL-10 production, whereas TNIL + LPS activated a strong response (Figure 2D). An interaction between the mannose moieties on gp120 and an MCLR(s) can therefore trigger IL-10 production from MDDCs from a significant proportion of human donors. The lack of effect of HA, which does not bind to DC-SIGN, compared to gp120 is consistent with the outcome of comparative immunization studies with these two viral receptor-binding glycoproteins in mice [11].

The blocking effect of the anti-DC-SIGN and anti-MR mAb combination implicated these MCLRs as likely mediators of the IL-10 response to gp120 (Figure 2A). Because this response is donor-dependent (Figure 1A), we measured DC-SIGN and MR expression on day-6 iMDDCs (the time of addition of gp120) from nine donors, as well as the expression of CD80, CD83, and CD86. DC-SIGN levels varied by 18-fold among these nine donors, MR by 3.3-fold (in studies of other sets of donors, we have found that the expression levels of both these receptors can vary by about an order of magnitude; unpublished data). The IL-10 response to JR-FL gp120 correlated with the level of DC-SIGN expression on day 6 (*n* = 9, *r^2^* = 0.52, *p* = 0.028) but not with MR expression (*n* = 9, *r^2^* = 0.19, *p* = 0.25). Moreover, there were no correlations between IL-10 production and the expression of CD80 (*n* = 9, *r^2^* = 0.052, *p* = 0.59), CD83 (*n* = 9, *r^2^* = 0.33, *p* = 0.14), or CD86 (*n* = 9, *r^2^* = 0.27, *p* = 0.19). Thus, of the five correlations with IL-10 production that we performed, the only substantial one was with DC-SIGN expression.

### HIV-1 gp120 Induces IL-10 Production via the ERK Signaling Pathway

**Figure 3:**
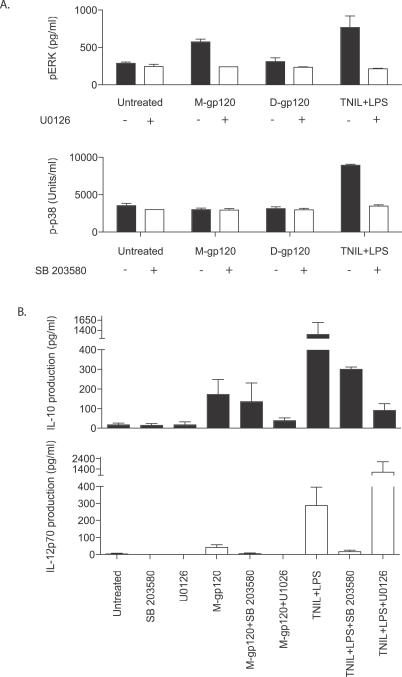
Involvement of the ERK1/2 and p38 MAP Kinase Signaling Pathways in the Induction of IL-10 and IL-12p70 by gp120 and TNIL + LPS (A) Day-6 MDDCs were incubated with or without JR-FL M-gp120 or D-gp120 (3 μg/ml), or with TNIL + LPS, for 10 min before pERK1/2 (upper panel) and p-p38 levels (lower panel) were measured by ELISA (black bars). The white bars show the effects of adding the pERK1/2 inhibitor UO126 (5 μM) or the p38 inhibitor SB 203580 (10 μM) 1 h before the gp120s or TNIL + LPS. The bars represent the mean values ± SEM for data derived from four different donors. (B) The experimental design was based on that used for (A), except that the iMDDCs were incubated with or without UO126 or SB 203580 for 1 h prior to the addition of M-gp120 or TNIL + LPS + CD40L and continued incubation for 24 h. IL-10 (upper panel) or IL-12p70 (lower panel) production was measured by ELISA after 24 h. The bars represent the mean values ± SEM for data derived from five different donors, which were not the same as the ones used in (A).

We next sought to identify which signaling pathway(s) was involved in the upregulation of IL-10 expression after the gp120-MCLR interaction. We focused on the ERK1/2 and p38 MAP kinase pathways [29], because ERK1/2 phosphorylation and activation promotes IL-10 production and inhibits IL-12 production by DC [30], whereas inhibition of ERK1/2 activation has the opposite effects [31]. Conversely, p38 mediates the induction of IL-12p70 expression by LPS [29]. Furthermore, DC-SIGN ligation by antibodies can lead to ERK1/2 phosphorylation [32]. We observed that TNIL + LPS strongly activated the phosphorylation of both ERK1/2 and p38 within 5 min, effects that persisted for 30–60 min (Figure 3A and unpublished data). A lesser, but still significant, level of ERK1/2 activation occurred after 5–10 min in MDDCs treated with M-gp120, ERK1/2 phosphorylation levels then declining back to baseline after 30 min. However, there was no detectable phosphorylation of p38 in response to M-gp120 at any time point (Figure 3A and unpublished data). D-gp120, in contrast, failed to activate the phosphorylation of either ERK1/2 or p38 (Figure 3A), implying that the gp120-MCLR interaction leads to the specific, albeit transient, activation of the ERK1/2 signaling pathway. In the same experiment, M-gp120, but not D-gp120, induced modest levels of IL-10 production, whereas TNIL + LPS + CD40L activated a much greater IL-10 response (Figure 3B), observations in proportion to the levels of ERK1/2 activation induced by the different stimuli (Figure 3A).

We used signaling inhibitors to determine whether there is a link between ERK1/2 activation and IL-10 production. MDDCs were treated with 5 μM U0126 (an ERK1/2 inhibitor) or 10 μM SB 203580 (a p38 MAP kinase inhibitor) for 1–2 h, then IL-10 and IL-12p70 production in response to JR-FL M-gp120 or TNIL + LPS + CD40L were measured 24 h later. U0126 inhibited ERK1/2 phosphorylation by ∼60% (Figure 3A), and the same compound reduced the IL-10 responses to both M-gp120 (by 70%) and TNIL + LPS + CD40L (by ∼90%) (Figure 3B). SB 203580 had a negligible effect on M-gp120-stimulated IL-10 production but did inhibit the IL-10 response to TNIL + LPS by 70% (Figure 3B).

In view of the reciprocal effects of the ERK1/2 pathway on IL-10 and IL-12 production by DCs [30,31], we also measured the IL-12p70 responses to M-gp120 and to TNIL + LPS. M-gp120 triggered a very slight increase in IL-12p70 expression. In contrast, TNIL + LPS activated a substantial IL-12p70 response that was completely blocked by SB 203580 but potentiated (4.7-fold) by U0126, a pattern consistent with a previous report [31]. Neither inhibitor, by itself, activated IL-10 or IL-12p70 production (Figure 3B).

Together, the use of signaling inhibitors implies that ERK1/2 activation is required for IL-10 production by MDDCs in response to either M-gp120 or TNIL + LPS.

### HIV-1 gp120 Impairs iMDDC Maturation

**Figure 4:**
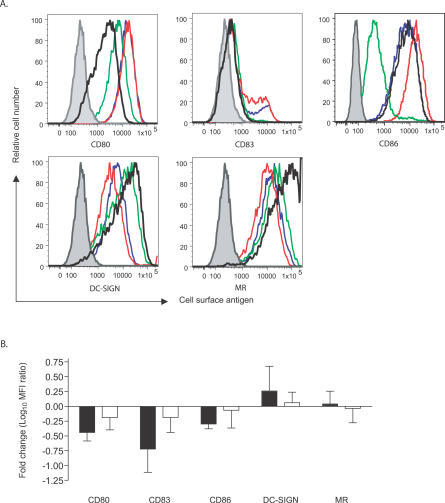
Gp120 Impairs iMDDC Maturation via Interaction with an MCLR(s) The maturation status of MDDCs was evaluated after treatment for 48 h (days 6–8) with TNIL + LPS + CD40L ± 3 μg/ml of JR-FL M-gp120 or D-gp120. The cell surface expression of CD80, CD83, CD86, DC-SIGN, and MR on CD11c+ cells was measured by flow cytometry as described in Supporting Information. (A) The histograms show expression of the surface markers on MDDCs from one donor whose expression marker response to gp120 was of average magnitude. The grey shaded profiles depict the use of isotype control mAbs, the other profiles were derived using the various specific test mAbs. The black curves represent control MDDCs; red curves, MDDCs treated with TNIL + LPS + CD40L; blue curves, TNIL + LPS + CD40L + D-gp120; green curves, TNIL + LPS + CD40L + M-gp120. (B) The average fold-changes (for 14 or 15 donors) in MFI values for MDDC cell-surface marker expression are depicted. The background MFIs obtained with the respective isotype controls were subtracted from MFIs for all conditions. Then the ratios of MFI for presence of gp120 over the MFI for absence of gp120 were calculated. For each marker, the MFI value derived from MDDC matured with TNIL + LPS + CD40L alone is thus defined as 1.0 (log ratio = 0). The means of the 10-logarithms of the ratios for all donors were calculated. The mean log MFI ratio ± SD for cells also treated with either M-gp120 (black bars) or D-gp120 (white bars) are plotted relative to this baseline value corresponding to TNIL + LPS + CD40L alone.

We used immunophenotypic analyses to investigate whether gp120 affects iMDDC maturation. Neither M-gp120 nor D-gp120 induced iMDDCs to mature in the absence of TNIL + LPS + CD40L; the cell-surface expression of no maturation marker changed by more than 1.5-fold (unpublished data). However, expression of CD80 was reduced by 3-fold, CD83 by 7-fold, and CD86 by 2-fold when iMDDCs were incubated with M-gp120 together with TNIL + LPS + CD40L, compared with when the cells were matured with TNIL + LPS + CD40L alone. DC-SIGN expression was 2- to 3-fold greater on MDDCs treated with TNIL + LPS + CD40L plus M-gp120 than on cells receiving only TNIL + LPS + CD40L, but MR expression was unchanged. D-gp120 did not mimic the effects of M-gp120 on the expression of CD80, CD83, CD86, and DC-SIGN, implicating an MCLR(s) as a mediator of these effects of gp120 (Figure 4). Furthermore, gp120 impaired the maturation of iMDDCs from both IL-10-responding and non-responding donors. The reduced expression of CD80, CD83, CD86, and the increased expression of DC-SIGN did not correlate with IL-10 secretion 48 h after gp120 addition among the 15 donors tested, of which nine were IL-10 responders, six non-responders. There were no correlations: *r^2^* = 0.05 for CD80 fold-decrease versus IL-10; *r^2^* = 0.02 for CD83 fold-decrease versus IL-10; *r^2^* = 0.00004 for the fold-increase in DC-SIGN expression versus IL-10. The reduction in CD86 expression also did not correlate with IL-10, *r^2^* = 0.00003.

The interaction of gp120 with an MCLR(s) therefore partially blocks the TNIL + LPS + CD40L-induced maturation of iMDDCs that normally leads to increases in CD80, CD83, and CD86 expression and a reduction in DC-SIGN expression. These events occur irrespective of whether the gp120-treated cells produce IL-10.

### HIV-1 gp120 Inhibits the Ability of mMDDCs to Induce T Cell Proliferation

We next explored whether the effects of gp120 on MDDC maturation (and cytokine production, see Supporting Information) would affect their ability to stimulate the proliferation of allogeneic T cells. To do this, M-gp120 or D-gp120 (JR-FL) was added to iMDDCs simultaneously with TNIL + LPS (i.e., on day 6 from the start of the MDDC culture). Influenza virus HA was used as a control antigen, also given simultaneously with TNIL + LPS. After the iMDDCs had been cultured with the various stimuli for 48 h, the cells were washed to remove any free gp120 or HA, then negatively selected for CD8, CD14, CD16, CD19, CD36, CD56, CD123, TCRγ/δ, and CD235α. CFSE-labeled, allogeneic CD4^+^ T cells were then added (the ratio of 1/10 was optimized for detection of T cell proliferation) for a 5-d co-culture (i.e., from days 8–13 from the start of the MDDC culture). T cell proliferation was measured as the proportion of CFSE-negative cells. Flow-cytometric histograms supporting the data presented below are shown in Figure S5.

**Figure 5:**
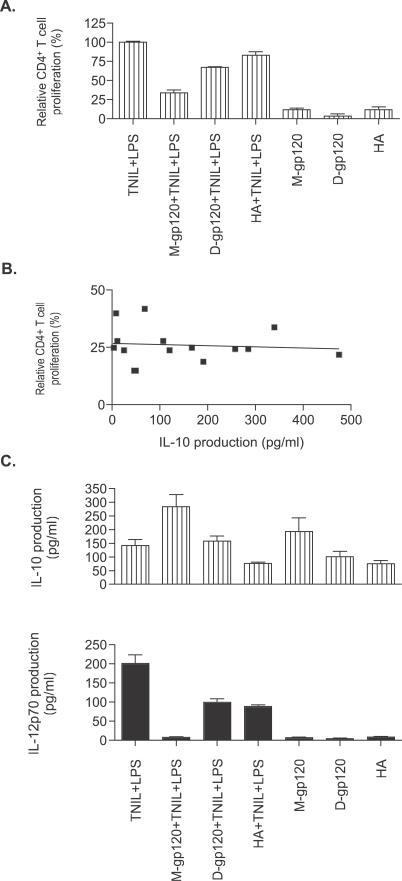
Treatment with gp120 Inhibits MDDC-Induced T Cell Proliferation (A) Day-6 MDDCs were incubated for 48 h with or without TNIL + LPS and/or JR-FL M-gp120, D-gp120, or influenza HA as specified on the *x*-axis. The MDDCs were then co-cultured for 5 d with CFSE-labeled CD4+ T cells before determination of the extent of the allogeneic mixed T lymphocyte reaction on day 13. Relative CD4^+^ T cell proliferation was calculated by first subtracting the background value for CFSE-negative cells obtained using unstimulated iMDDCs (9.95% ± 0.70%, *n* = 15) from the value obtained when the MDDCs were stimulated with TNIL + LPS (46.4% ± 1.54%, *n* = 15). The net value was defined as 100% and used for normalization. The bars represent the mean values ± SD for data derived from 15 donors (except for influenza HA; ten donors) tested in 15 independent experiments. The superantigen SEB served as a positive control for CD4+ T cell stimulation in the absence of any MDDCs; CFSE dilution in response to SEB was 130% ± 2.9% (*n* = 15) of that seen with TNIL + LPS (unpublished data). (B) The extent of T cell proliferation in co-cultures (as in A) containing iMDDCs exposed to M-gp120 + TNIL + LPS + CD40L is plotted as a function of the IL-10 response of the iMDDCs to M-gp120 after 24 h. There was no correlation for iMDDCs from 15 donors (*r^2^* = 0.0008). (C) In a subset of the experiments shown in (A), extracellular cytokine levels were measured at the end of the MDDC-T cell co-culture (i.e., on day 13). The bars represent the mean values ± SEM from five different donors. Upper panel, IL-10; lower panel, IL-12p70.

MDDCs treated with gp120 in the absence of TNIL + LPS did not stimulate T cell proliferation (Figure 5A). However, exposing the TNIL + LPS-stimulated MDDCs to M-gp120 for 24–48 h reduced their ability to stimulate T cell proliferation by ∼65%. D-gp120 was less inhibitory, the ∼30% decrease being little different from the ∼20% decrease seen with the HA control antigen. M-gp120 depressed proliferation significantly more than did D-gp120 (one-tailed Mann-Whitney U test, *n* = 15, *p* < 0.0001). MDDCs from all 15 donors tested (ten IL-10 responders, five non-responders) behaved similarly in the T cell proliferation assay; the relative proliferation of CD4^+^ T cells in co-cultures with M-gp120 + TNIL + LPS-treated MDDCs varied in a narrow range (60%–85% reduction in proliferation) over a broad range of IL-10 responses (0–420 pg/ml) (Figure 5B). Overall, there was no correlation between IL-10 levels in the cultures of the gp120-treated, TNIL + LPS-stimulated MDDCs on day 8 and the inhibition of subsequent T cell proliferation (% CFSE dilution versus IL-10, *r^2^* = 0.0008). The lack of effect of IL-10 is not surprising because both cytokines and any stimulus for their continued secretion are washed out of the MDDC cultures before the T cells are added. However, the principal point is that, as with maturation marker expression, the MDDC phenotype is adversely affected by gp120 treatment, whether or not the cells have an immediate IL-10 response.

We also found that JR-FL and KNH1144 gp120s each caused a ∼70% reduction in the capacity of LPS + TNIL-stimulated MDDCs from four donors to induce T cell proliferation (similarly to what is shown for JR-FL gp120 in Figure 5A, unpublished data). Since KNH1144 gp120 does not induce IL-10 secretion from MDDCs (Figure 1C), this experiment corroborates the finding that the anti-proliferative effect is independent of IL-10 from MDDCs (Figure 5B). It also confirms that KNH1144 gp120 is biologically active in the MDDC system despite its inability to induce IL-10 expression.

We measured the concentrations of both IL-10 and IL-12p70 in the various MDDC-T-cell co-cultures on day 13 (Figure 5C). IL-10 concentrations varied by <5-fold overall, the co-cultures with MDDCs exposed to M-gp120 + TNIL + LPS containing the highest level (280 ± 45 pg/ml). The IL-10 response to M-gp120 + TNIL + LPS was significantly higher than to D-gp120 + TNIL + LPS (one-tailed Mann-Whitney U test, *n* = 5, *p* = 0.028). IL-12p70 concentrations varied much more substantially. They were very low (<10 pg/ml) in co-cultures containing MDDCs treated with M-gp120, D-gp120, or HA in the absence of TNIL + LPS. When TNIL + LPS was used to mature the MDDCs, IL-12p70 concentrations reached 200 ± 22 pg/ml. The inclusion of D-gp120 or HA caused a modest (∼2-fold) reduction, but when M-gp120 was used only baseline levels of IL-12p70 were produced (7.2 ± 1.7 pg/ml). The IL-12p70 response to M-gp120 + TNIL + LPS was significantly lower than to D-gp120 + TNIL + LPS (one-tailed Mann-Whitney U test, *n* = 5, *p* = 0.0040). Thus, compared to the use of TNIL + LPS alone, exposure of the MDDCs also to M-gp120 caused a 76-fold increase in the IL-10/IL-12p70 ratio in the co-cultures, whereas the use of D-gp120 and HA caused only 2.4- and 1.3-fold increases, respectively. Moreover, the pattern of IL-12p70 responses in the various co-cultures (Figure 5C, lower panel) was similar to the pattern of T cell proliferation in the same cultures (Figure 5A). IL-4 was also measured, the concentrations ranging from 5–15 pg/ml in the different co-cultures, with no obvious pattern of response detectable (unpublished data).

In conclusion, MDDCs matured in the presence of gp120 are functionally impaired, irrespective of whether they secrete IL-10 soon after gp120 binds to MCLRs.

## Discussion

We show here that exposure to HIV-1 gp120s can impair the maturation of human iMDDCs, triggering cells from some donors to secrete IL-10, a cytokine generally associated with immunosuppressive responses [23]. Irrespective of whether they secrete IL-10, the gp120-treated MDDCs mature inefficiently in response to conventional stimuli, and their abilities to stimulate the proliferation of T cells in co-cultures are impaired. The latter defect could be due to their reduced expression of CD80, CD83, and CD86 and hence a weakening of the co-stimulatory interactions with T cells that drive the latter's proliferation. The reduction in IL-12p70 levels (and a substantial increase in the IL-10/IL-12p70 ratio) in the co-cultures may also be relevant [33].

These various effects are a consequence of an interaction between the mannose components of gp120 glycans and an MCLR(s), in that the enzymatic removal of mannoses from gp120 reduced or prevented their occurrence. We also probed the IL-10 response to gp120 using various blocking ligands. Thus, CV-N and the 2G12 mAb bind to gp120 mannoses, and each inhibited IL-10 induction, whereas inhibitors of gp120 binding to CD4, CCR5, or CXCR4 were ineffective. Furthermore, mannan, another MCLR ligand, activated IL-10 expression. Also relevant is that gp120 induces IL-10 expression in immunized mice [11]: Gp120 cannot bind to murine CD4, CCR5, or CXCR4, or to the murine MCLR with the greatest sequence similarity to human DC-SIGN [34]. However, five murine DC-SIGN homologues have been described [35], so it is possible that some of them do bind gp120. The influenza HA Env protein does not induce IL-10 expression either in the immunized mice or in our own in vitro experiments; HA binds the MR [36] but not DC-SIGN or DC-SIGNR [37].

Several different MCLRs are known or potential binding sites for gp120 on DC, including DC-SIGN, langerin, and the MR [38]. We found that mAbs to DC-SIGN and the MR together completely ablated the IL-10 response to gp120, while the anti-DC-SIGN mAb was partially inhibitory by itself. Furthermore, there was a correlation between the extent of IL-10 production and the level of DC-SIGN expression on the MDDCs. Together, these observations strongly suggest a role for DC-SIGN binding in the IL-10 response to gp120 but other MCLRs, particularly the MR, also seem likely to be involved.

M-gp120, but not its demannosylated derivative, activated ERK1/2 phosphorylation, and the ERK1/2 inhibitor U0126 inhibited the IL-10 response to M-gp120. These findings imply that the gp120-MCLR interaction triggers the ERK1/2 signaling pathway and that this is necessary for activation of IL-10 expression. Whether the same pathway mediates the other MDDC responses to gp120 remains to be determined.

This conclusion is consistent with earlier reports on the role of the ERK1/2/MAP kinase pathways in the IL-10 response when DCs are activated by other stimuli, including TLR ligands and DC-SIGN-specific antibodies [30–32]. The binding of pathogens, including HIV-1, to DC-SIGN has also been shown to activate the Raf-1-acetylation-dependent signaling pathway [39]. The gp120-treated MDDC from about half the 71 donors we studied secreted elevated amounts of IL-10, and the response pattern was consistent when 11 donors were re-tested a month later. Hence, genetic or other invariable factors and not, for example, an inter-current infection seem most likely to determine whether a donor's MDDCs respond to gp120 in this way, or not. Complex host genetic factors influence IL-10 gene regulation [23,40–42], suggesting one area for further study. The genetics of MCLR expression might also be relevant; different MCLRs might be involved to different extents on MDDCs from different donors. DC-SIGN expression varies considerably in rectal tissue samples from different individuals and has been associated with local increases in the IL-10/IL-12 ratio [33]. Nonetheless, the IL-10 response to gp120 is only one marker for the adverse effect of this ligand on MDDCs; whether or not a donor's cells secreted IL-10 in response to gp120, they were functionally impaired, matured poorly, and were unable to efficiently stimulate T cell proliferation.

We also observed that both the concentration and the identity of the gp120 protein influenced the IL-10 response. Two of the three tested gp120s (JR-FL and LAI) triggered IL-10 release from MDDCs of responsive donors, whereas gp120 from KNH1144 did not. We do not yet know why KNH1144 differs from the other two gp120s in this regard. The most likely explanation is that there are subtle differences between the gp120s in exactly how they interact with one or more MCLR, and that the IL-10 response is particularly sensitive to a specific, but as yet uncharacterized, facet of these interactions. Differences in how diverse HIV-1 virions and gp120 proteins interact with DC-SIGN have been reported, although the variations in gp120 structure that affect the interaction have still to be fully defined [43] (M. Jansson, personal communication). A sequence alignment of the JR-FL, LAI, and KNH1144 gp120 proteins, with emphasis on the positions of N-linked glycans, suggests a number of potentially relevant differences (Figure S6). Since understanding the molecular basis for the lack of IL-10 induction might help in the design of new Env-based immunogens, mutagenesis studies that focus on the N-linked glycans of JR-FL and KNH1144 could be informative, as might the use of additional gp120s that vary in sequence and that are expressed in cell types that lead to differences in glycosylation patterns. It is important, however, that any such reagents be highly purified free of the LPS contaminants that are common in most commercial gp120 preparations and in some others made under non-GMP conditions. The same constraints apply to the use of inactivated HIV-1 virions. We have not yet tested virions, as the focus of the present study is on soluble Env proteins that are (or were) vaccine candidates. Moreover, virions contain several TLR activators that might induce different cytokine responses that complicate any analysis of the effects of the Env component [44–46].

Several earlier studies have shown that gp120 and inactivated HIV-1 virions can have complex effects on MDDCs and their interactions with T cells and on cytokine secretion by both cell types in vitro. Thus, compared to LPS, R5, and X4 gp120s both stimulated much less IL-12 production from MDDCs, but without IL-10 release [19]. Just as we have observed, gp120-treatment impaired MDDC maturation in response to classical stimuli, reducing their ability to stimulate T cells, but unlike our results, CD80, CD83, and CD86 were up-regulated on the gp120-treated cells [19]. In another study, exposure of MDDCs to X4 gp120 up-regulated CD80 and CD86 and down-regulated MR, with increased secretion of IL-10, IL-12, IL-18, and TNF-α [47]. Various surface markers were also up-regulated on HIV-1-infected MDDCs, associated with an inability of the cells to secrete IL-12 in response to CD40L [48]. The receptor interactions of gp120 most responsible for its various biological effects were not determined in these various studies. Gp120 is also known to stimulate IL-10 release from monocyte/macrophages in vitro [17,18,20,22,24]. There is one report that MDDCs undergoing continued stimulation with GM-CSF and IL-4 did not secrete IL-10 in response to gp120, although differences in the experimental conditions are probably responsible, and the donor- and gp120-dependent variation we now describe may also be relevant [49].

Our observations are consistent with a mounting body of evidence on the biological effects of ligating MCLRs on DCs. Thus, HIV-1 BaL and a specific DC-SIGN mAb have recently been shown to activate Rho-GTPase-dependent signals via DC-SIGN that favor the formation of DC-T-cell synapses and HIV-1 infection of the T cells [50]. The same signaling events also induced the ATF3 transcription factor that suppressed TLR-response genes, attenuating the LPS responses of the cells by reducing IL-12p70 secretion and down-modulating CD86 and HLA-DR. Thus, as we observed with gp120, the anti-DC-SIGN mAb induced a semi-immature state in the MDDCs, which failed to stimulate T cell proliferation effectively [50]. Indeed, the binding of an antibody to DC-SIGN was previously found to activate ERK-1/2 but not p38 [32], similar to what we have observed with gp120. Cross-linking the MR via a specific mAb can have a broadly similar effect on the MDDC phenotype [51]. It will be worth studying whether the downstream signals activated by the MCLR MAbs are also triggered by gp120. Of further note is that an allergenic glycoprotein from peanuts also induces ERK1/2 signaling in MDDCs via DC-SIGN, but up-regulates MHC and co-stimulatory molecules and thereby increases the ability of the MDDCs to activate T cell proliferation [52]. Thus, there may be considerable subtleties to how different glycoproteins and mAbs bind to DC-SIGN and other MCLRs on the MDDC surface, and the intracellular consequences of these interactions. Most mAbs to DC-SIGN or the MR do not induce transmembrane signals, but some do [50,51]; likewise, some gp120s induce IL-10 expression, others do not. One relevant point may relate to how an MCLR ligand is mannosylated: adding O-linked mannoses to ovalbumin increases lympho-proliferation in mixed BMDC-T-cell cultures while N-linked mannoses have the opposite effect, and mannosylated ovalbumin impaired IL-12p70 secretion [53]. Most mannose residues on gp120 are N-linked [54], but the relative amounts of N- and O-linked moieties could vary between strains and influence the overall signaling patterns that are activated. Other pathogens also use mannose moieties to suppress immune responses, again via binding to MCLRs. For example, the *M.Tb* cell wall component ManLAM binds to DC-SIGN at a similar site to gp120′s, induces IL-10 production, impairs DC maturation, and suppresses the host immune response to this pathogen [55,56]. Some lactobacilli do much the same, although without the involvement of mannose residues [57].

Although DC-SIGN, and MCLRs in general, are important sentinels for the presence of pathogens, some organisms may be able to subvert at least some of the natural functions of these receptors for their own purposes [58]. DC-SIGN, in particular, may be considered as an unconventional PRR (pattern recognition receptor) that drives T_H_2 and T_reg_ responses [32,58]. Silencing SOCS-1 in DC has been shown to reduce the suppressive effect of gp120 on the production of pro-inflammatory cytokines in vitro [59]. Mice immunized with gp120-pulsed, SOCS-1-silenced DC produced higher and more sustained titers of anti-gp120 antibodies, and T_H_1-polarized cellular responses to gp120 [59]. Conversely, over-expressing SOCS-3 in murine DC increased IL-10 expression, and SOCS-3-transduced DC primed a T_H_2-dominant response when co-cultured with CD4^+^ T cells in vitro [60]. Perhaps these observations are linked mechanistically to ours?

Caution must always be taken when extrapolating from cell culture systems to the more complex environment of tissues in vivo where the DC phenotype differs from the MDDCs we have used here and where gp120 concentrations are hard to estimate [16,61]. We note, however, that DCs and T cells isolated from HIV-1-infected persons can have aberrant phenotypes that are broadly similar to those of the gp120-exposed MDDCs that we have studied in vitro [62]. In particular, elevated numbers of tolerogenic semi-mature DCs, and FOXP3^+^ CD4^+^ regulatory T cells, have been observed in lymph nodes of HIV-1-infected people [63]. Moreover, high levels of IL-10, accompanied by a reduction in IL-12, can be found in plasma during primary HIV-1 infection [64]. IL-10 can have a substantial effect on the course of viral infections [65]. Thus, blocking IL-10 signaling by antibodies to its receptor promotes the clearance of lymphocytic choriomenigitis virus and prevents the establishment of a persistent infection [66,67]. Perhaps similar events are involved in persistent infection by HIV-1? Thus it is possible that, during primary infection, *env*-gene products could help suppress the development of anti-HIV-1 immune responses at this critical time, particularly as virion-associated gp120 is more efficient than free gp120 at inducing various signaling events [68]. If so, the retention of high mannose moieties on the Env complex would be yet another defense HIV-1 uses in its battle with host immunity. We previously noted that the presence of mannoses on Env is paradoxical because they might facilitate virion clearance from the blood [25]: Counter-functions would justify their retention.

Our observations could help understand the outcome of immunizing with Env-based antigens, and perhaps why different individuals respond to these vaccines with Ab titers that can vary over a several-log range [5–9]. When milligram amounts of gp120 are delivered in a bolus into tissues, local concentrations are likely to be high enough to affect the performance of various immune system cells, including DCs, during the earliest, formative stages of the immune response [16]. In a comparative DNA and protein immunization study in mice, the antibody and cytokine responses to gp120 were strongly T_H_2-polarized, whereas responses to HA were T_H_1-biased. Furthermore, the T_H_2 bias of the anti-gp120 response did not occur in IL-10 knock-out mice [11]. Although T-helper phenotypes are more complex in humans than mice, the responses to gp120, during infection and after vaccination, do appear to be T_H_2-biased [12–15]. Including Env in multi-component HIV/SIV vaccines can sometimes be deleterious to protection [69,70]. Also, immunizing horses with insect cell–expressed Env proteins (which are enriched for high-mannose moieties) from Equine Infectious Anemia Virus (EIAV) enhanced post-immunization infection with EIAV, whereas EIAV Env proteins expressed in mammalian cells induced protective responses [71–73]. Insect cell–expressed gp120 proteins were also comparatively poor immunogens in mice, because of a limited ability to induce T-helper responses [9].

Any vaccine-related, adverse influences of the high-mannose moieties on gp120 glycans could be overcome by treating gp120 with a mannosidase enzyme. We are now investigating whether this strategy improves the immunogenicity of HIV-1 Env proteins. Deleting a subset of N-linked glycans altered the IgG isotype profile of the antibody response to the HCV E1 protein in immunized mice and improved its immunogenicity overall [74]. Of course, raising higher titers of antibodies and/or reducing the rate of decay of the antibody response to HIV-1 Env will achieve little if those antibodies are non-neutralizing. Our hope, however, is that a general increase in the immunogenicity of Env proteins could facilitate the development of otherwise sub-threshold NAb responses, and/or enable lower amounts of Env trimers to be used. Combining the mannose-removal technique with other strategies intended to increase the immunogenicity of NAb epitopes should also be possible. Several other vaccine antigens that are considered to be problematic from the immunogenicity perspective, such as RSV F, RSV G, CMV gB, and Ebola GP, are also highly glycosylated and/or can bind to MCLRs (S. Plotkin and B. Graham, personal communication) [75–77]. Whether these proteins might also contain high-mannose moieties or other carbohydrate structures that can interact with MCLRs and that could be removed enzymatically is worth considering.

## Materials and Methods

### Recombinant proteins and cytokines.

Recombinant, CHO-cell expressed monomeric gp120s from HIV-1 JR-FL, LAI, and KNH1144 were manufactured at Progenics, as previously described, under GMP conditions [78]. The concentration of the gp120 stocks was 1 mg/ml, with Endotoxin contamination <3 EU/ml. Gp120 was added to target cells at 3 μg/ml (25 nM), except when otherwise specified. Insect cell–expressed influenza hemagglutinin (HA) protein (100 μg/ml) was purchased from Protein Sciences Corporation and used at 3 μg/ml (Endotoxin <10 EU/ml, no fungal or bacterial contamination)**.**

LPS from *Salmonella Typhimurium* (1 mg, Sigma) was used at 100 ng/ml. Recombinant soluble CD40L (50 μg, Bristol-Myers Squibb) with an Endotoxin level of < 0.1 ng per μg (1 EU/μg) was used at 1 ng/ml; TNF-α and IL-1β (R&D Systems) at 25 ng/ml and 10 ng/ml, respectively.

### Inhibition of gp120-induced IL-10 production.

iMDDCs were incubated for 1 h at 37 °C with various agents before gp120 was added. The anti-DC-SIGN mAb AZN-D1 (Beckman Coulter), the isotype control mouse IgG1 (Clone 2T8-2F5, Beckman Coulter), the anti-mannose receptor (MR; CD206) mAb Clone 15–2 (Cell Sciences), and the isotype control mouse IgG1, κ (Clone MOPC-21, BD Pharmingen) were each used at 40 μg/ml, alone or in combination. The CCR5 inhibitor AD101 (from J. Strizki, Schering Plough Research Institute)[79] and the CXCR4 inhibitor AMD3100 (from G. Bridger, AnorMed Incorporated) [80] were each used at 10 μM. Mannan (Sigma) was added at 30 μg/ml.

Alternatively, gp120 was mixed with sCD4 (Progenics) [81], mAb b12 (from D. Burton, Scripps) [82], mAb 2G12 (from H. Katinger, University of Vienna) [83], each at 25 μg/ml, or with cyanovirin-N (CV-N; from R. Shattock, St. George's, London) [26] at 5 μg/ml for 1 h at room temperature on a roller before addition to the cells.

### Mannosidase treatment of recombinant gp120.

The mannose residues were removed from JR-FL gp120 to make demannosylated gp120 (D-gp120) as follows [25]. Aliquots of gp120 (120 μg) were incubated for 16–18 h at 37 °C with no enzyme (mock treatment; M-gp120) or with α-(1,2,3,6)-mannosidase (Jack Bean, GKX-5010; 25 Units/mg, 0.14 Units/μg gp120; from ProZyme Incorporated) in a final volume of 1.2 ml, in the presence of protease inhibitors (Roche). A control incubation of enzyme-only (no gp120) was also performed. The samples were desalted into half-strength PBS (1/2 PBS) using PD-10 desalting columns (GE Healthcare) and concentrated to 1 ml using Vivaspin 30k MWCO 6 ml spin concentrators (Vivascience). After addition of 1 volume of 1/2 PBS, each sample was processed using the Endofree Red 5/1 Endotoxin removal kit (Profos AG). The final volumes of the D-gp120 and M-gp120 preparations after endotoxin removal were ∼2 ml, with endotoxin levels <8–20 EU/mg and gp120 concentrations 60 μg/ml. SDS-PAGE and western blot analyses were performed using mAbs 2G12 and CA13 (ARP3119).

### ELISA for gp120-binding ligands.

gp120 proteins were captured onto ELISA wells via sheep antibody D3724 to the gp120 C-terminus, and mAb or CD4-IgG2 binding was assessed essentially as described previously [84]. For DC-SIGN binding to the captured gp120, the standard procedure was adapted as follows: The plates were washed three times with TSM (20 mM Tris, 150 mM NaCl, 1 mM CaCl_2_, 2 mM MgCl_2_), followed by incubation with TSM/1% BSA for 30 min. After three washes with TSM, DC-SIGN-Fc (a gift from T. Geijtenbeek [85]) in TSM was added for 2 h, with or without a prior incubation for 15 min with EGTA (10 mM) or mAb AZN-D1 (10 μg/ml). The plates were washed five times with TSM/0.05% Tween, then bound DC-SIGN-Fc was detected with peroxidase-labeled goat anti-human Fc (1:3,000) in TSM/0.05% Tween using standard conditions.

### Cell culture.

Peripheral blood mononuclear cells (PBMC) were isolated from buffy coats (New York Blood Center or Research Blood Components) by Ficoll density gradient centrifugation. Monocytes were isolated to high purity (>98%) by magnetic cell sorting with anti-CD14-coated beads according to the manufacturer's recommendations (Miltenyi Biotec). The percentage of CD14^+^ monocytes among the cells sorted from PBMC was determined by flow cytometry and always exceeded 98%. The CD14^−^ fraction was frozen and used as the source of T cells for MDDC-T cell co-cultures. The monocytes were subsequently cultured for 6–8 d in complete culture medium (RPMI 1640, GIBCO/Invitrogen) containing 1 mM sodium pyruvate, 0.1 mM nonessential amino acids, 2 mM L-glutamine, 25 mM HEPES, 100 U/ml penicillin, 100 μg/ml Streptomycin (all obtained from GIBCO/Invitrogen), and supplemented with 5% Human AB serum (Sigma) (R-5), 1,000 U/ml GM-CSF (Leukine, Sargramostim), and 1,000 U/ml of recombinant human IL-4 (R&D Systems) at 37 °C in an atmosphere containing 5% CO_2_. Every 2 d, 400 μl of medium were gently removed from each well and replaced by 500 μl of fresh medium containing the appropriate cytokines.

### MDDC maturation.

iMDDCs were either used without maturation or were differentiated for 24 h or 48 h with TNIL + LPS ± CD40L, a mixture of inflammatory cytokines: 25 ng/ml of TNF-α and 10 ng/ml of IL-1β (TNIL), and LPS (10 ng/ml or 100 ng/ml) ± CD40L (1 μg/ml). Because elevated CD83 expression on MDDCs (a response to TNF-α) is necessary but not sufficient for IL-12 responses [86], CD40L, a strong inducer of IL-12, was included in all experiments in which IL-12p70 was measured. The flow-cytometric analysis of maturation markers is described in Supporting Information.

### Reverse transcriptase-PCR.

iMDDC were incubated with and without gp120 (3 μg/ml) for various times at 37 °C, and analyzed for the expression of IL-10 mRNA by reverse transcriptase (RT)-PCR. Total RNA was extracted from 1 × 10^6^ iMDDCs by using the Absolutely RNA Miniprep Kit (Stratagene) according to the manufacturer's manual. The isolated total RNA (2 μl) was used for synthesis of cDNA using the Super Script III First-Strand Synthesis System for RT-PCR ( Invitrogen). Human IL-10 and β-actin transcripts were amplified using the following primers: IL-10 forward 5′-ATGCCCCAAGCTGAGAACCAAGACCCA-3′ and reverse 5′-TCTCAAGGGGCTGG GTCAGCTATCCCA-3′. The PCR product is 352 bp and was verified by sequencing. The β-actin primers used were: forward 5′- TCCTGTGGCATCCACGAAACT-3′ and reverse 5′-GAAGCATTTGCGGTGGACGA T-3′. Their amplification product of 315 bp was also verified by sequencing. The annealing temperature for gradient PCR detection of IL-10 transcripts was optimized so as to avoid cross-reaction with IL-4, IL-6, IL-12p35, and IL-12p40.

### Cytokine or chemokine measurements.

Purified monocytes were cultured in RPMI 1640 supplemented with 5% human AB serum, 1,000 U/ml GM-CSF, and 1,000 U/ml IL-4 for 6 d in order to produce iMDDCs, then washed thoroughly. The cells were aliquoted at various densities from 5 × 10^5^ to 1 × 10^6^ cells/ml into 24-well plates, and then stimulated as described in Results. Cytokine IL-10 and IL-12p70 concentrations in cell-free culture supernatants were measured by ELISA using OptEIA kits from BD Pharmingen, as per the manufacturer's protocol. The detection sensitivity for each cytokine was 4 pg/ml. Chemokine CCL17/TARC, CCL22/MDC, CCL19/MIP-3β, and CXCL10/IP10 were measured by ELISA assays using DuoSet ELISA kits from R&D Systems.

### MAPK assay.

For analysis of MAPK signaling pathways, day-6 iMDDCs were collected, washed three times with warm PBS, and then cultured in a serum-free medium for at least 24 h before additional stimuli. The cells were then incubated in the presence or absence of gp120 or TNIL + LPS for various times. Where indicated, an MEK inhibitor (U0126, 5 μM) or a p38 inhibitor (SB 203580, 10 μM) was added to the cultures 1–2 h before gp120 or TNIL + LPS. The cells were harvested and washed twice with cold PBS, then centrifuged into a pellet, and resuspended in 300 μl of lysis buffer (1% Nonidet P-40, 0.1% SDS, 0.5% sodium deoxycholate in PBS) containing PMSE (100 μg/ml) and a protease inhibitor mixture (500 μg/ml) (Roche Diagnostics). In some experiments, the supernatants were also collected and stored at −80 °C for later analysis of cytokine content. The total protein concentration of the cell pellets was measured using the bicinchoninic acid assay (Pierce). Samples containing 30 μg of total protein were heated at 100 °C for 5 min in the presence of DTT, then the following assay kits were used according to the manufacturer's instructions (Calbiochem): p38[TOTAL] ELISA kit; P38[pTpY180/182] ELISA kit; ERK1/2[TOTAL] ELISA kit; ERK1/2 [pTpY185/187] ELISA kit.

### T cell proliferation assay.

Allogeneic CD4^+^ T cells were obtained by negative selection with magnetic beads and washed twice with PBS (see Supporting Information); the cells were then incubated with 2.5 μM carboxy-fluorescein diacetate, succinimidyl ester (CFSE) (derived from a 5-mM CFSE stock; Molecular Probes) for 15 min at room temperature, with gentle agitation every 2–3 min [87]. The reaction was quenched by the addition of an equal volume of RPMI 1640 containing 10% human AB serum followed by incubation for 5 min. The cells were then washed with PBS three times and resuspended at 2 ×10^6^ cells/ml in complete culture medium before use in experiments. For the mixed T lymphocyte reaction assay, CFSE-labeled or unlabeled allogeneic CD4^+^ T cells were co-cultured with differentially treated MDDCs at a 1/10 ratio for 5 d. (In preliminary experiments, the DC:T cell ratio was varied over the range 1/10^−2^ to 1/10^2^ in 10-fold increments, for both iMDDCs and mMDDCs, the optimal ratio for detecting T cell proliferation after 5 d of co-culture being 1/10.) Proliferation of the CFSE-labeled naïve T cells was analyzed by flow cytometry [87]. Supernatants were collected from the co-cultures of MDDCs with unlabeled allogeneic CD4^+^ T-cells on day 5, for measurement of cytokine levels by ELISA.

### Statistical analysis.

IL-10 measurements were subjected to the D'Agostino and Spearman omnibus normality test. The data were not uniformly normal. Hence, differences between groups were analyzed by one-tailed Mann-Whitney U test. The α level was set to 0.05. Correlations rather than regression analyses were performed since we analyzed measured variables (IL-10 secretion, cell surface antigen expression, and cell proliferation).

### Supporting information.

 The derivation and phenotypic characterization of the iMDDCs and mMDDCs, as well as the purification of CD4^+^ T cells, are described in Text S1. We also show the time course of IL-10 induction and Ab controls for the blocking of gp120-induced IL-10 secretion. Furthermore, we describe the effects of mAbs to DC-SIGN and MR on the expression of MDDC maturation markers, and provide examples of flow cytometric histograms illustrating inhibition of T cell proliferation. The cytokine and chemokine responses of gp120-treated MDDC are discussed.

## Supporting Information

Figure S1 Flow-Cytometric Analysis of Surface Markers on PBMC, Monocytes, and MDDCs (488 KB PDF)

Figure S2 Time Course of IL-10 Production Induced by JR-FL gp120 (12.9 MB PDF)

Figure S3 Effect of Anti-MR and Anti-DC-SIGN mAbs on MDDC Maturation (540 KB PDF)

Figure S4 Effect of Anti-MR and Anti-DC-SIGN mAbs and Other Ligands on IL-10 Production by MDDCs (797 KB PDF)

Figure S5 Gp120 Impairs iMDDC Maturation via Interaction with an MCLR(s) (257 KB PDF)

Figure S6 Alignment of gp120 Amino Acid Sequences (318 KB PDF)

Text S1 Supporting Information (163 KB PDF)

## Abbreviations

DC: dendritic cell
Env: envelope glycoprotein
iMDDC: immature monocyte-derived dendritic cell
LPS: lipopolysaccharide
MCLR: mannose C-type lectin receptor
MDDC: monocyte-derived dendritic cell
mMDDC: mature monocyte-derived dendritic cell
MR: mannose receptor
NAb: neutralizing antibody
RT: reverse transcriptase
TNIL: TNFα and IL-1β
