# Supplementary material for: HIV-1 gp120 Mannoses Induce Immunosuppressive Responses from Dendritic Cells
Source: PLoS Pathog. 2007 Nov 2;3(11):e169. doi: 10.1371/journal.ppat.0030169 (PMC2048530; doi:10.1371/journal.ppat.0030169)
Supplement: Supplementary file 1 [file ppat.0030169.sd001.pdf]

Supporting Information

HIV-1 gp120 Mannosylated Glycans Induce Immunosuppressive Responses from Dendritic Cells

Meimei Shan (1), Per Johan Klasse (1) , Kaustuv Banerjee (1),
Antu K. Dey (1), Sai Prasad N. Iyer (2), Robert Dionisio (1), Dustin Charles (1),
Lila Campbell-Gardener (1), William C. Olson (2), Rogier W. Sanders (3),
John P. Moore (1,*)

(1) Dept. of Microbiology and Immunology, Weill Medical College of Cornell University, New York, New York, USA
(2) Progenics Pharmaceuticals Inc, Tarrytown, New York, USA
(3) Laboratory of Experimental Virology, Dept. Medical Microbiology, Center of Infection and Immunity Amsterdam (CINIMA), Academic Medical Center of the University of Amsterdam, Amsterdam, The Netherlands

*Address correspondence to John P. Moore, Dept. of Microbiology and Immunology, Weill Medical College of Cornell University, 1300 York Avenue, Box 62, New York, New York 10021, USA, email: jpm2003@med.cornell.edu

Supporting Information

Derivation and properties of pure MDDC populations

Surface antigens were analyzed by flow cytometry on days 6 and 8 of culture, before and after treatment of the cells as described in the text. The cells were first washed twice with PBS containing 0.5% human AB serum, then incubated for 20 min at 4°C with different combinations of mAbs. The following mAbs were from BD Pharmingen: FITC-conjugated mAbs to CD3 (Clone HIT3a, Cat No. 555339) or HLA DR (Clone G46-6, Cat No. 555811); Per CP-conjugated mAb to CD4 (Clone SK3, Cat No. 347324); PE-conjugated mAbs to CD14 (Clone M5E2, Cat No. 555398), CD80 (Clone L307.4, Cat No. 340294), CD86 (Clone IT2.2, Cat No. 555665) or CD206 (Clone 19.2, Cat No. 555954); APC-conjugated mAb to CD11c (Clone S-HCI-3, Cat No. 340544). The following mAbs were from Bioscience: FITC-conjugated mAb to DEC-205 (Clone MG38, Cat No. 12-2-59). The Clone 120507 mAb (Cat No. FAB 161P) to CD209 (DC-SIGN) was from R&D Systems. Each mAb was diluted so as to optimize the immunofluorescence signal. After washing three times with cold PBS/human AB serum, fluorescence events were acquired with an LSR II analyzer (BD Pharmingen), and the data were analyzed using Flowjo (Tristar Inc, Ashland, OR).

The blood monocytes used to generate MDDC were typically 98-99% pure, as indicated by CD14 expression, <5% of freshly isolated monocytes from any donor expressed CD86, HLA DR, and DEC 205 and none expressed CD80, CD83, DC-SIGN or MR expression on the cell surface (Figs.S1A, S1B). After cultivation with GM-CSF and IL-4 for 6 days, a high proportion of the cells expressed CD11c, DC-SIGN, MR and

CD86, but few were positive for CD80 and CD83. This pattern of surface expression markers is consistent with the vast majority of cells having the immature MDDC (iMDDC) phenotype. Thus, the phenotypic analyses of the MDDC cultures confirm that the IL-10 secretion we observe is not derived from monocytes/macrophages, but from MDDC.

Likewise, in phase-contrast light microscopy the vast majority of the cells were spherical, larger than monocytes and had short dendrites; i.e. their morphology was typical of iMDDC (not shown).

On day 6, iMDDC were collected, washed, and matured with TNIL+LPS+CD40L for an additional 48h before phenotypic analysis. The expression of CD80, CD83, and CD86 was up-regulated, and that of MR and DC-SIGN was down-regulated (Fig.S1C).

We also studied the time course of gp120-induced IL-10 secretion. Extracellular IL-10 levels peaked 24-48h after addition of JR-FL gp120 (Fig.S2, upper panel), whereas IL-10 mRNA transcripts could first be detected after 5h, peaked at ~10h and gradually declined thereafter (Fig.S2, bottom panel).

Effects of gp120 on MDDC cytokine and chemokine profiles

We measured the release of several pro-inflammatory cytokines and chemokines from MDDC treated for 24h with JR-FL gp120. Unlike TNIL+LPS-treatment, gp120 did not induce the expression of IL-6, IL-8, TNF-α, IL-1β, CXCL10 or CCL19, but it did activate secretion of CCL22 and CCL17 (data not shown), chemokines that are known to act on T_H2 effector cells and regulatory T cells [1]. Furthermore, the lack of CXCL10 and CCL19 induction by gp120 was notable, since the former chemokine is a ligand for CXCR3, a receptor preferentially expressed on Th1 cells, while the latter interacts with

CCR7 and plays a well-established role in the recruitment of DC precursors and T cells in secondary lymphoid organs [2,3]. The secretion of IL-6 and IL-8 was rarely observed, and it was not associated with the IL-10 response in the same 11 repeat donors (data not shown). Overall, then, treatment of MDDC with gp120 induced a chemokine/cytokine profile that would eventually favor the recruitment of T_H2 and Treg cells while negatively regulating a T_H1 type immune response.

MCLR antibodies do not by themselves affect MDDC phenotype

The various MCLR-blocking mAbs and their isotype controls, either alone or in combination, did not cause significant cell death during a 48h incubation with iMDDC, and they did not themselves induce MDDC maturation as measured by the expression of CD80, CD83 and CD86 (Fig.S3). However, the anti-MR and anti-DC-SIGN mAbs, either alone or in combination, down-regulated the TNIL+LPS+CD40L-stimulated expression of CD80 (anti-MR by 6.7%; anti-DC-SIGN by 44%; anti-MR and anti-DC-SIGN in combination by 72%) and CD83 (anti-MR by 63%; anti-DC-SIGN by 88%; anti-MR and anti-DC-SIGN in combination by 92%). CD86 expression was affected to a lesser extent (<30%) (Fig.S3).

There were no significant differences in cell death (as measured by gating on forward and side scatters) among the untreated or TNIL+LPS-treated MDDCs, either in the absence or presence of the anti-MR and/or anti-DC-SIGN mAbs. Hence the complete blockade of gp120-induced IL-10 expression by the combination of mAbs to MR and DC-SIGN (Fig.2A) is not attributable to a non-specific (i.e., toxic) effect of the antibodies on the cells.

MCLR antibodies and other inhibitors do not by themselves affect IL-10 production

iMDDC were incubated for 1h at 37°C with various agents before gp120 was added. The anti-DC-SIGN mAb AZN-D1, the isotype control mouse IgG1 Clone 2T8-2F5, the anti-MR mAb Clone 15-2 and the isotype control mouse IgG1, κ Clone MOPC-21 were each used at 40μg/ml, alone or in combination. None of those antibodies induced IL-10 production in MDDC by itself, and none blocked IL-10 production induced by TNIL+LPS. When the CCR5 inhibitor AD101 and the CXCR4 inhibitor AMD3100 were each used at 10μM, neither blocked gp120-induced IL-10 production. As controls for the experiment shown in Fig.2A (main text), the gp120 ligands sCD4, mAb b12, mAb 2G12 (each at 25μg/ml) and CV-N (5μg/ml) were incubated for 1h at room temperature before addition to the cells. None of them induced IL-10 production (Fig.S4).

Gp120 impairs iMDDC maturation via interaction with an MCLR(s)

Histograms showing T-cell proliferation in co-cultures with MDDCs treated in various ways are presented in Fig.S5.

Purification of $CD4^+$ T cells

$CD4^+$ T cells were purified by the use of the $CD4^+$ T cell Isolation Kit II (Cat No. 130-091-155, Miltenyi Biotec) in accordance with the manufacturer's instructions. In brief, $CD4^+$ T helper cells were isolated by depletion of other cells (negative selection). Non-$CD4^+$ T cells were labeled with a cocktail of biotin-conjugated monoclonal antibodies against CD8, CD14, CD16, CD19, CD36, CD56, CD123, TCR-gamma/delta and Glycophorin A; the cells were then incubated with an anti-biotin MAb conjugated to magnetic MicroBeads. When the cell suspension was passed through a column, $CD4^+$ T

cells were eluted pure, while labeled cells were retained by the magnetic field of the MACS Separator.

Alignment of gp120 sequences

We inspected the primary sequences of gp120s from JR-FL, LAI and KNH1144, to see if there was any obvious reason why the first two stimulate IL-10 production from MDDCs via MCLRs, whereas the last does not (Fig.1C). The three sequences are aligned in Supplementary Figure 6, with differences in the N-linked glycans highlighted. There are five positions at which a glycan could be present in JR-FL but absent from KNH1144, and two converse possibilities. Because the determinants of how gp120 interacts with DC-SIGN and other MCLRs are incompletely understood, it is not possible to state with any certainty which of the glycans absent from KNH1144 are most likely to be relevant to its inability to induce an IL-10 response from MDDCs. Moreover, it is possible that sequence differences that do not directly eliminate one or more glycan sites from KNH1144 gp120 indirectly affect the geometry of an MCLR binding site(s). Another scenario is that KNH1144 gp120 does interact with the relevant MCLR(s) on the surface of MDDCs, but in an orientation that does not cause transduction of the signals that activate the IL-10 response. A series of mutagenesis studies based on the differences between the JR-FL and KNH1144 gp120 sequences will be required to identify the underlying biological mechanism.

We used McVector 7.2.3 HIV/SIV Sequence Locator Tool available via the Los Alamos Database (http://www.hiv.lanl.gov/content/hiv-db/LOCATE/locate.html) for the alignment, which is based on the LAI numbering.

Supporting Information References

1. McIlroy A, Caron G, Blanchard S, Fremaux I, Duluc D, et al. (2006) Histamine and prostaglandin E up-regulate the production of Th2-attracting chemokines (CCL17 and CCL22) and down-regulate IFN-gamma-induced CXCL10 production by immature human dendritic cells. Immunology 117: 507-516.
2. Chieppa M, Bianchi G, Doni A, Del Prete A, Sironi M, et al. (2003) Cross-linking of the mannose receptor on monocyte-derived dendritic cells activates an anti-inflammatory immunosuppressive program. J Immunol 171: 4552-4560.
3. Lebre MC, Burwell T, Vieira PL, Lora J, Coyle AJ, et al. (2005) Differential expression of inflammatory chemokines by Th1- and Th2-cell promoting dendritic cells: a role for different mature dendritic cell populations in attracting appropriate effector cells to peripheral sites of inflammation. Immunol Cell Biol 83: 525-535.

Figures for Supporting Information

Figure S1. Flow-cytometric analysis of surface markers on PBMC, monocytes and MDDC

- **A.** The top panel shows forward and side scatter plots of PBMC isolated from a healthy blood donor. The density of cells is indicated by color-coding: blue, lowest to red, highest. The lower population of cells has the characteristics of live lymphocytes (47% of all cells), the upper population corresponds to monocytes (12%). The middle forward-side scatter plot shows the purity (82%) of the monocytes after positive selection with anti-CD14-conjugated beads. The lower diagram depicts double cell-surface staining with antibodies to CD4 and CD14; ~99% of the cells were $CD14^+$.
- **B.** The scatter plots show the cell-surface expression (y-axis) on $CD11c^+$ MDDCs (x-axis) of HLA-DR, CD86, CD83 and CD80; and of DC-SIGN, MR and DEC-205.
- **C.** Monocytes purified as in **A** were cultured for 6 days with GM-CSF and IL-4 to generate iMDDC (upper panel), and then analyzed for co-expression of CD11c and CD80, CD83, CD86, MR and DC-SIGN. After 6 days of culture, the iMDDC were further matured by TNIL+LPS for additional 48h to generate mMDDC (bottom panel). The expression of the same markers was then analyzed by flow cytometry.

Figure S2. Time course of IL-10 production induced by JR-FL gp120

- **A.** The IL-10 concentrations in MDDC cultures are shown as a function of time after addition of JR-FL gp120 (3μg/ml). The data points show mean values ± SD (the

error bars lie within the symbols) of duplicate ELISA determinations. The three curves represent the responses from three different donors.

B. The ethidium bromide-stained agarose gel shows gp120- or TNIL+LPS-stimulated IL-10 mRNA expression in MDDC from a single donor. Untreated MDDC were negative for IL-10 mRNA at any time-point (not shown). The β-actin band (315bp) confirms that mRNA recovery was consistent at the different time points.

Figure S3. Effect of anti-MR and anti-DC-SIGN mAbs on MDDC maturation

As controls for the blocking experiments, we studied the effect of anti-MR and anti-DC-SIGN mAbs on iMDDC maturation in the presence and absence of the TNIL+LPS+CD40L stimulus. On day 6, iMDDC were collected, washed, and resuspended at a concentration of $1x10^6$ cells/ml. The cells were preincubated with different test antibodies and isotype controls (listed on the x-axis) for 1 h at 37°C. Without further washing, TNIL+LPS was added, or not, as indicated on the x-axis, and the culture was continued for 48h. The anti-DC-SIGN mAb AZN-D1, its isotype control, the anti-MR mAb Clone 15-2 and its isotype control, were used at 40μg/ml each, alone or in combination. The bars represent the MFI values for CD80, CD83 and CD86, as indicated. The data shown were derived from one of two similar experiments.

Figure S4. Effect of anti-MR and anti-DC-SIGN mAbs, and other ligands on IL-10 production by MDDCs

To determine whether the anti-MR and anti-DC-SIGN mAbs, and gp120 ligands, inhibit gp120-induced IL-10 secretion (main-text Fig.2A) via a direct blockade of receptor interactions, we performed control experiments involving the same agents on their own or with a TNIL+LPS stimulus replacing gp120. The MDDCs were prepared as in Fig.S3 (and in main-text Fig.2A), then treated as indicated on the x-axis. The anti-DC-SIGN mAb AZN-D1, its isotype control, the anti-MR mAb Clone 15-2 and its isotype control were used at 40μg/ml each, alone or in combination. The other agents listed on the horizontal axis are detailed in the Methods and in the Supporting Information text. The upper panel shows the lack of effect of small molecule co-receptor ligands on IL-10 induction by JR-FL gp120, and the inability of various gp120 or receptor ligands to activate IL-10 expression in the absence of gp120. The response to TNIL+LPS is also shown for comparison. In the lower panel, the lack of blocking effects of various mAbs on IL-10 induction by TNIL+LPS alone is depicted. The bars represent the mean values ± SEM for IL-10 secretion from MDDC from three to five different donors.

Figure S5. Gp120 impairs iMDDC maturation via interaction with an MCLR(s).
The histograms show T-cell proliferation observed in cultures with MDDCs from one of the donors used to derive the mean data shown in Fig.4A of the main article. The MDDC were treated as indicated under each histogram. The numbers in horizontal bars inside the histograms represent the percentage of CFSE dilution.

Figure S6. Alignment of gp120 amino-acid sequences

The amino-acid sequence of JR-FL gp120 is shown, as well as the differences between it and the sequences of LAI and KNH1144 gp120s. N-linked glycan sites are colored red, with the ones present in JR-FL but not KNH1144 sequences highlighted by black arrows above the sequence, and those present in KNH1144 but not JR-FL indicated by open arrows below the sequence.
