# Supplementary material for: HIV-1 gp120 Mannoses Induce Immunosuppressive Responses from Dendritic Cells
Source: PLoS Pathog. 2007 Nov 2;3(11):e169. doi: 10.1371/journal.ppat.0030169 (PMC2048530; doi:10.1371/journal.ppat.0030169)
Supplement: Supplementary file 2 [file ppat.0030169.sg001.pdf]

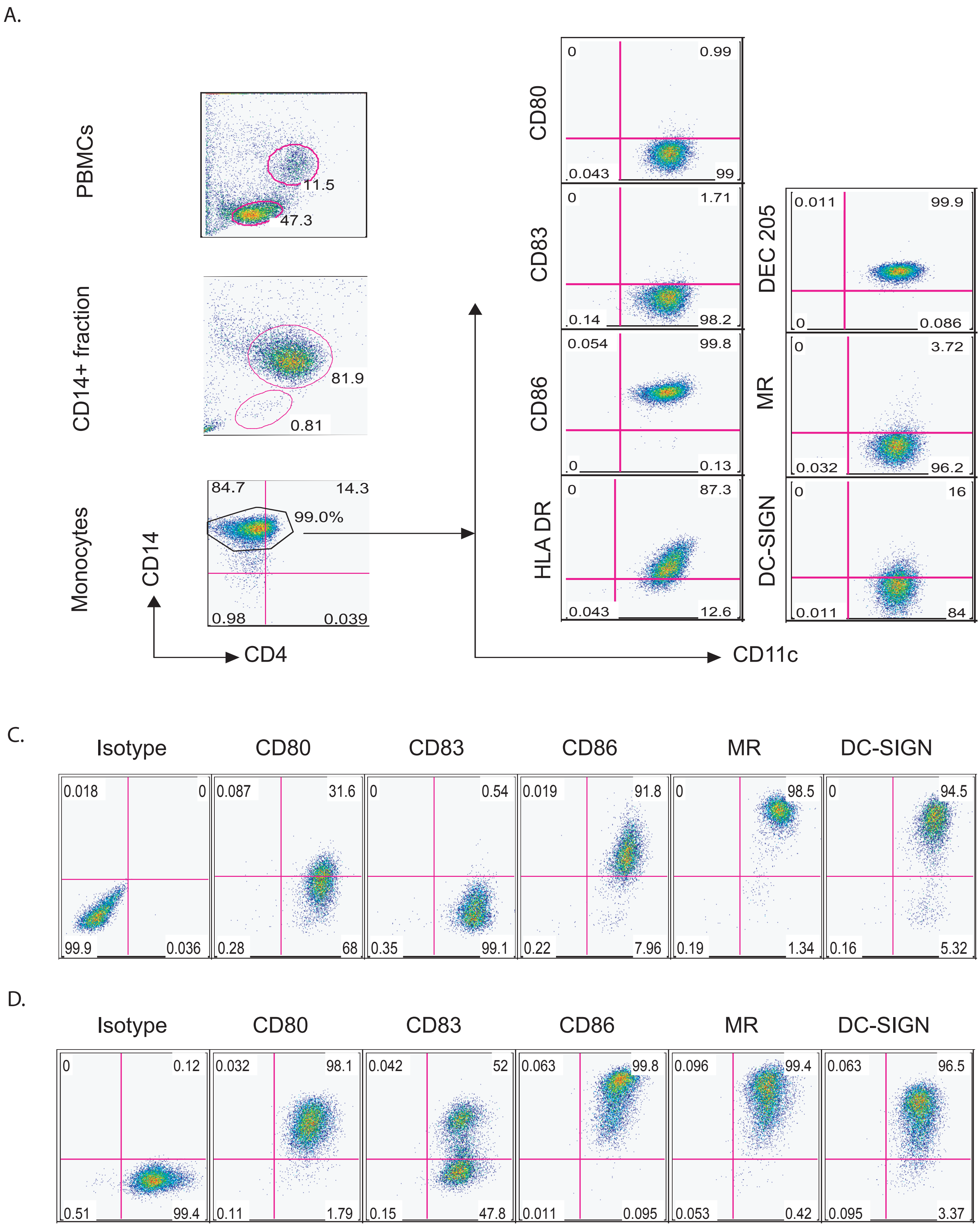

A.
PBMCs
11.5
47.3
CD14+ fraction
81.9
0.81
Monocytes
84.7
14.3
99.0%
0.98
0.039
CD14
CD4
CD80
0
0.99
0.043
99
CD83
0
1.71
0.14
98.2
CD86
0.054
99.8
0
0.13
HLA DR
0
87.3
0.043
12.6
DEC 205
0.011
99.9
0
0.086
MR
0
3.72
0.032
96.2
DC-SIGN
0
16
0.011
84
CD11c
C.
Isotype
0.018
0
99.9
0.036
CD80
0.087
31.6
0.28
68
CD83
0
0.54
0.35
99.1
CD86
0.019
91.8
0.22
7.96
MR
0
98.5
0.19
1.34
DC-SIGN
0
94.5
0.16
5.32
D.
Isotype
0
0.12
0.51
99.4
CD80
0.032
98.1
0.11
1.79
CD83
0.042
52
0.15
47.8
CD86
0.063
99.8
0.011
0.095
MR
0.096
99.4
0.053
0.42
DC-SIGN
0.063
96.5
0.095
3.37
