# Supplementary material for: HIV-1 gp120 Mannoses Induce Immunosuppressive Responses from Dendritic Cells
Source: PLoS Pathog. 2007 Nov 2;3(11):e169. doi: 10.1371/journal.ppat.0030169 (PMC2048530; doi:10.1371/journal.ppat.0030169)
Supplement: Supplementary file 3 [file ppat.0030169.sg002.pdf]

A.

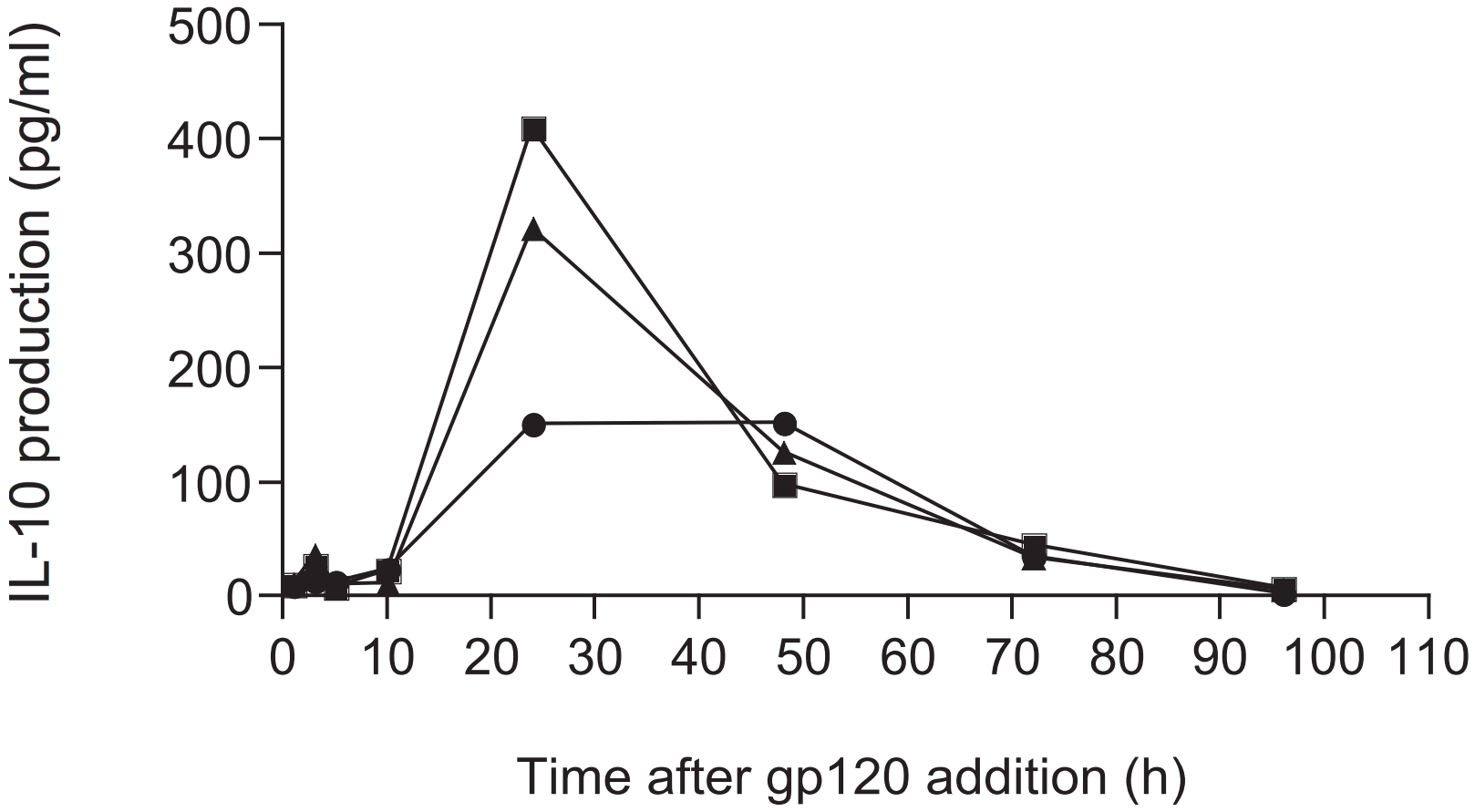

IL-10 production (pg/ml)
500
400
300
200
100
0
0
10
20
30
40
50
60
70
80
90
100
110
Time after gp120 addition (h)

B.

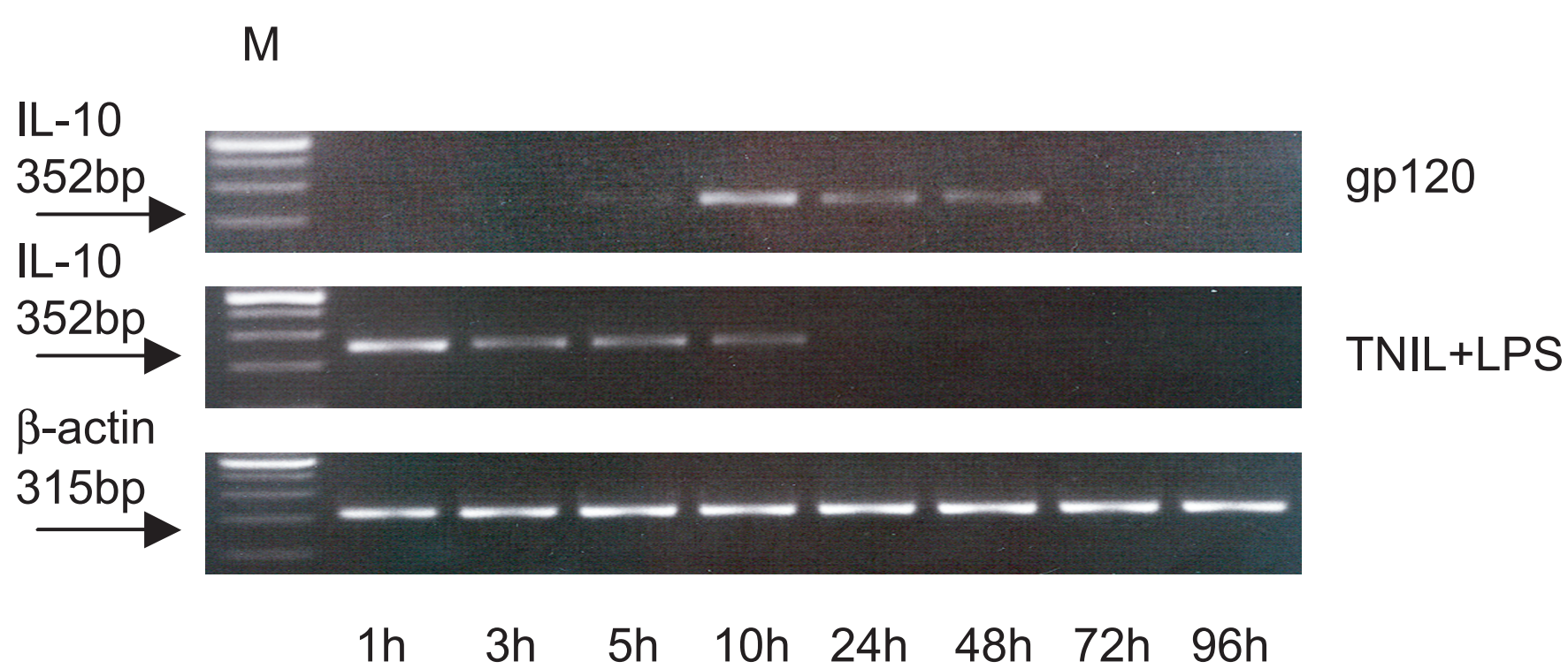

M
IL-10
352bp
gp120
IL-10
352bp
TNIL+LPS
β-actin
315bp
1h
3h
5h
10h
24h
48h
72h
96h
