# Supplementary material for: HIV-1 gp120 Mannoses Induce Immunosuppressive Responses from Dendritic Cells
Source: PLoS Pathog. 2007 Nov 2;3(11):e169. doi: 10.1371/journal.ppat.0030169 (PMC2048530; doi:10.1371/journal.ppat.0030169)
Supplement: Supplementary file 4 [file ppat.0030169.sg003.pdf]

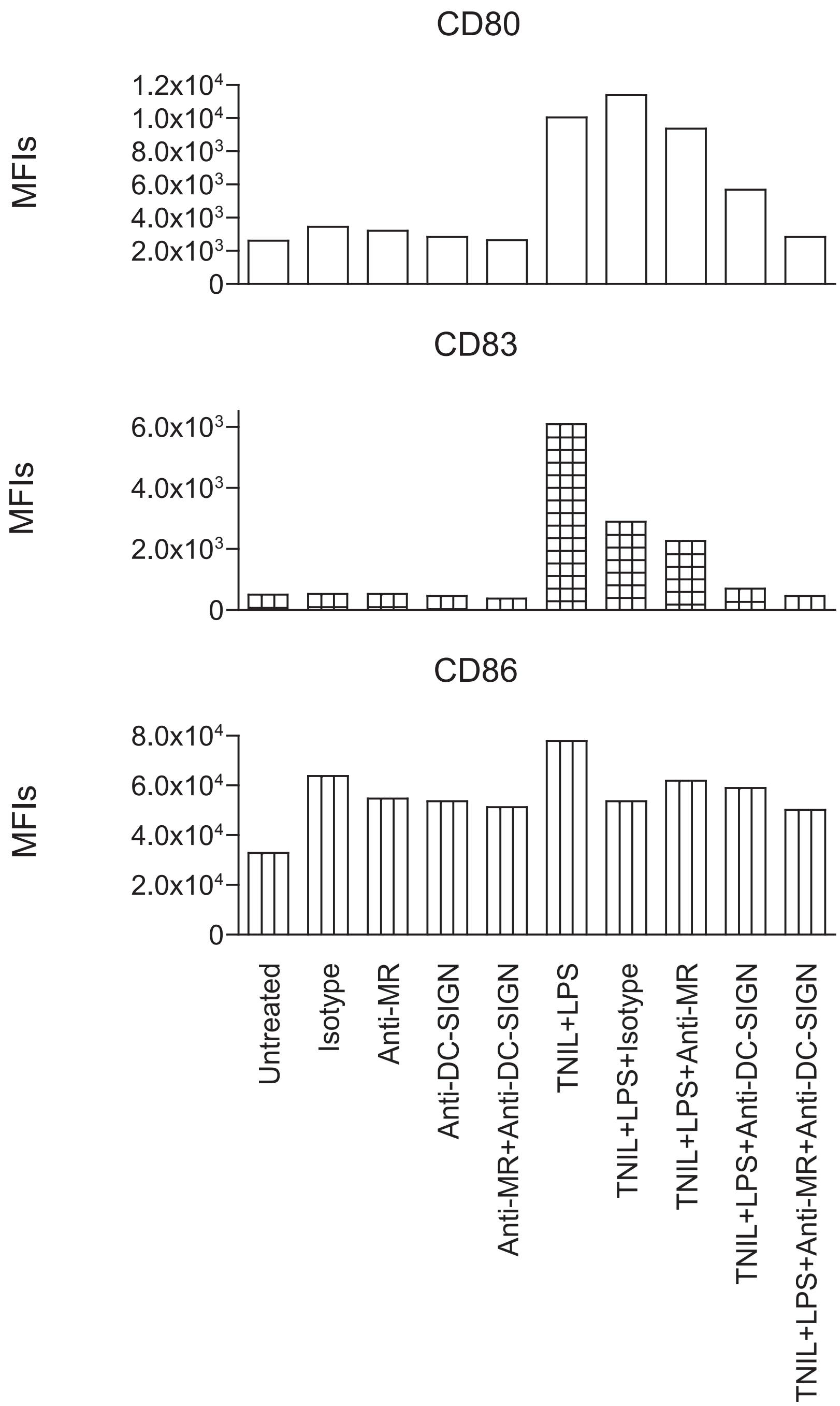

CD80
MFIs
1.2x10^4
1.0x10^4
8.0x10^3
6.0x10^3
4.0x10^3
2.0x10^3
0
CD83
MFIs
6.0x10^3
4.0x10^3
2.0x10^3
0
CD86
MFIs
8.0x10^4
6.0x10^4
4.0x10^4
2.0x10^4
0
Untreated
Isotype
Anti-MR
Anti-DC-SIGN
Anti-MR+Anti-DC-SIGN
TNIL+LPS
TNIL+LPS+Isotype
TNIL+LPS+Anti-MR
TNIL+LPS+Anti-DC-SIGN
TNIL+LPS+Anti-MR+Anti-DC-SIGN
