# Supplementary material for: HIV-1 gp120 Mannoses Induce Immunosuppressive Responses from Dendritic Cells
Source: PLoS Pathog. 2007 Nov 2;3(11):e169. doi: 10.1371/journal.ppat.0030169 (PMC2048530; doi:10.1371/journal.ppat.0030169)
Supplement: Supplementary file 5 [file ppat.0030169.sg004.pdf]

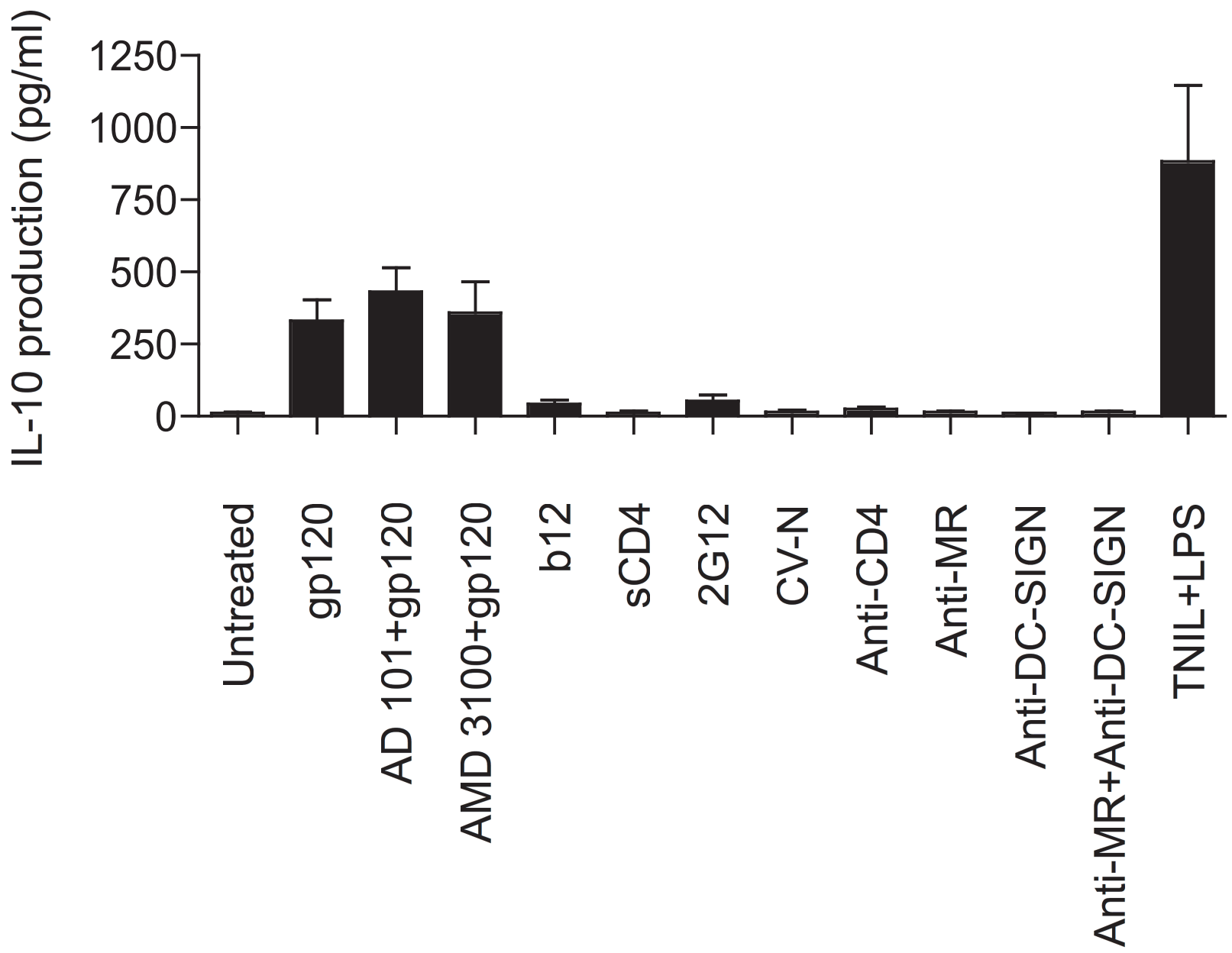

IL-10 production (pg/ml)
1250
1000
750
500
250
0
Untreated
gp120
AD 101+gp120
AMD 3100+gp120
b12
sCD4
2G12
CV-N
Anti-CD4
Anti-MR
Anti-DC-SIGN
Anti-MR+Anti-DC-SIGN
TNIL+LPS

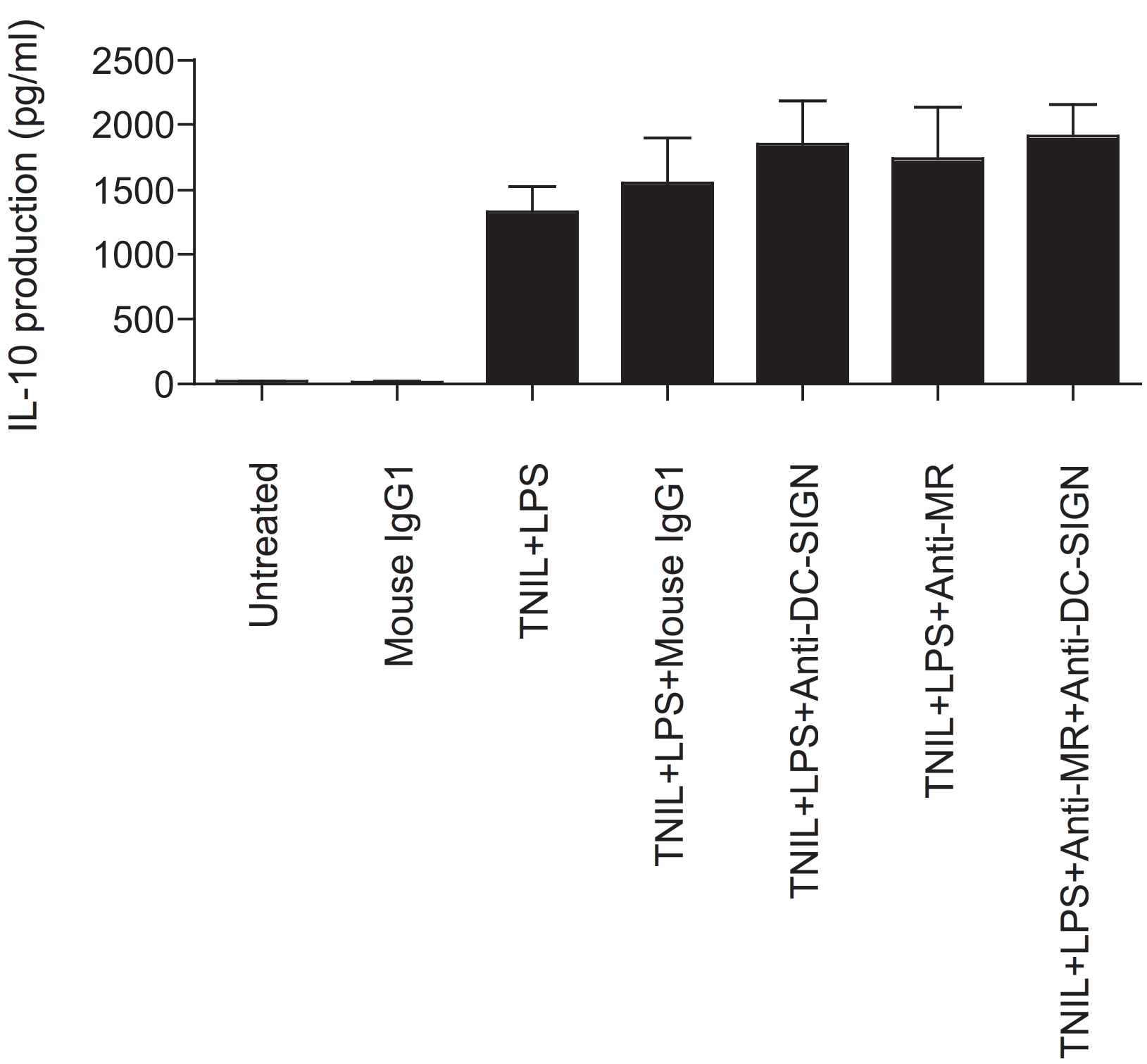

IL-10 production (pg/ml)
2500
2000
1500
1000
500
0
Untreated
Mouse IgG1
TNIL+LPS
TNIL+LPS+Mouse IgG1
TNIL+LPS+Anti-DC-SIGN
TNIL+LPS+Anti-MR
TNIL+LPS+Anti-MR+Anti-DC-SIGN
