# Supplementary material for: HIV-1 gp120 Mannoses Induce Immunosuppressive Responses from Dendritic Cells
Source: PLoS Pathog. 2007 Nov 2;3(11):e169. doi: 10.1371/journal.ppat.0030169 (PMC2048530; doi:10.1371/journal.ppat.0030169)
Supplement: Supplementary file 6 [file ppat.0030169.sg005.pdf]

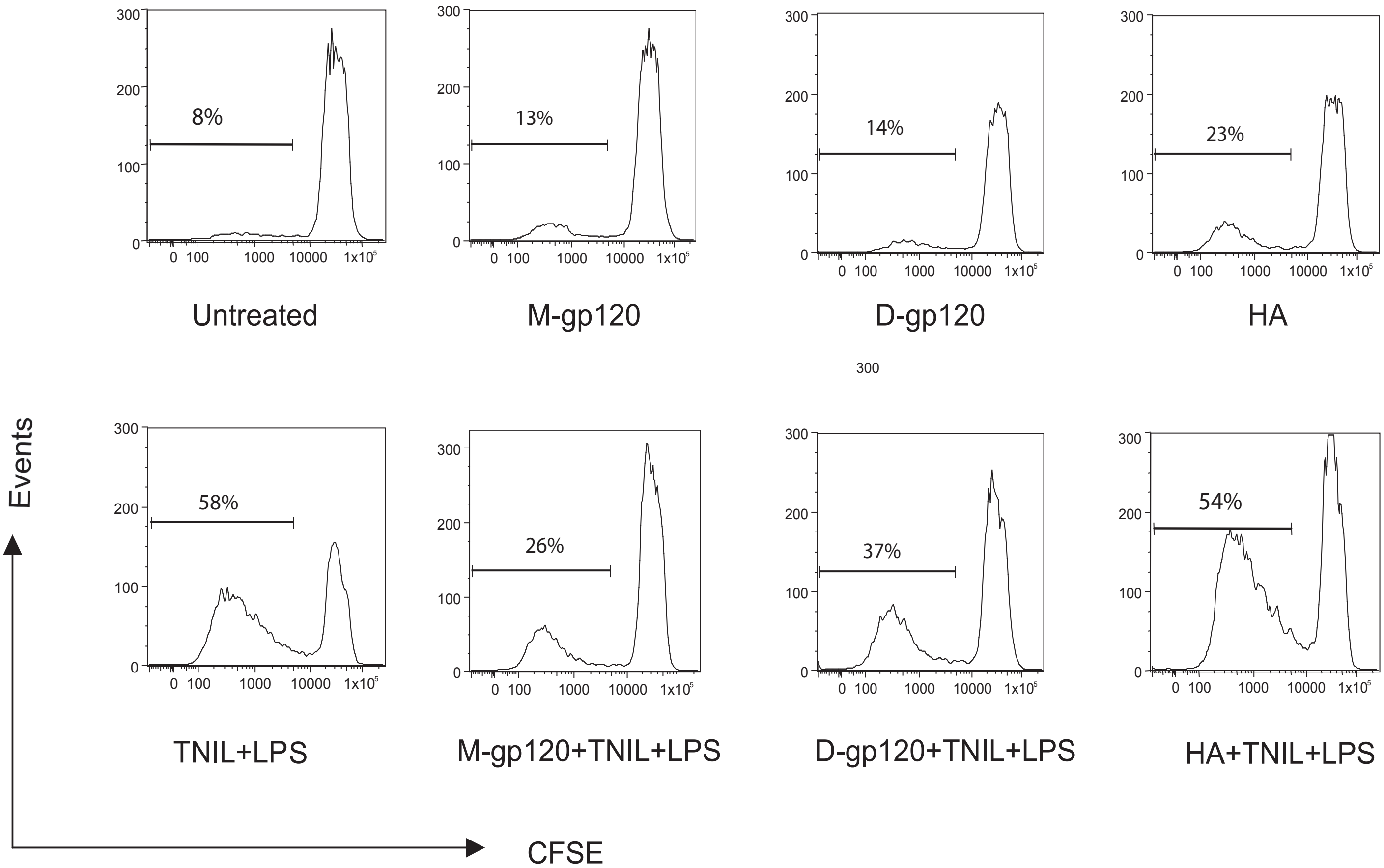

8%
13%
14%
23%
Untreated
M-gp120
D-gp120
HA
58%
26%
37%
54%
TNIL+LPS
M-gp120+TNIL+LPS
D-gp120+TNIL+LPS
HA+TNIL+LPS
Events
CFSE
