# Supplementary material for: HIV-1 gp120 Mannoses Induce Immunosuppressive Responses from Dendritic Cells
Source: PLoS Pathog. 2007 Nov 2;3(11):e169. doi: 10.1371/journal.ppat.0030169 (PMC2048530; doi:10.1371/journal.ppat.0030169)
Supplement: Supplementary file 7 [file ppat.0030169.sg006.pdf]

```
                                                                     50
JR-FL  :  MRVKGIRKSYQYLWK-G---GTLLLGILMICSAVEK-LWVTVYYGVPVWKEATTTLFCASDAKAYDTEVHNVWATHACVPTDPNPQE
LAI    :  MRVK---EKYQHLWRWGWKWGTMLLGILMICSATEK-LWVTVYYGVPVWKEATTTLFCASDAKAYDTEVHNVWATHACVPTDPNPQE
KNH1144:  MRVMGIQMNCQHLLRWG----TMILGLIIICNAVNSNLWVTVYYGVPVWKDAETTLFCASDAKAYKTEKHNVWATHACVPTDPNPQE

                    100                                                  150
JR-FL  :  VVLENVTEHFNMWKNNMVEQMQEDIISLWDQSLKPCVKLTPLCVTLNCKDVNATNTTNDSEGTM-------ERGEIKNCSFNITTSIRD
LAI    :  VVLVNVTENFNMWKNDMVEQMHEDIISLWDQSLKPCVKLTPLCVSLKCTDLGNATNTNSSNTNSSSGEMMMEKGEIKNCSFNISTSIRG
KNH1144:  IPLENVTEEFNMWKNKMVEQMHTDIISLWDQSLQPCVKLAPLCVTLNCTDVTNVTDVSGTRGNITIMKEM--EGEIKNCSFNMATEIRD

                                     200
JR-FL  :  EVQKEYALFYKLDVVPIDNNNTSYR------LISCDTSVITQACPKISFEPIPIHYCAPAGFAILKCNDKTFNGKGPCKNVSTVQCT
LAI    :  KVQKEYAFFYKLDIIPIDNDTTSYT------LTSCNTSVITQACPKVSFEPIPIHYCAPAGFAILKCNNKTFNGTGPCTNVSTVQCT
KNH1144:  KKQKVYSLFYRLDVVPINQGNSSSKNSSEYRLISCNTSAITQACPKVSFEPIPIHYCAPAGFAILKCRDKEFNGTGECKNVSTVQCT

          250                                 300
JR-FL  :  HGIRPVVSTQLLLNGSLAEEEVVIRSDNFTNNAKTIIVQLKESVEINCTRPNNNTRKSIHI--GPGRAFYTTGEIIGDIRQAHCNISR
LAI    :  HGIRPVVSTQLLLNGSLAEEEVVIRSANFTDNAKTIIVQLNQSVEINCTRPNNNTRKSIRIQRGPGRAFVTIGKI-GNMRQAHCNISR
KNH1144:  HGIKPVVSTQLLLNGSLAEEKVKIRTENITNNAKTIIVQLVEPVRISCTRPNNNTRESVRI--GPGQAFFATGDIIGDIRQAHCNVSR

                    350                                                   400
JR-FL  :  AKWNDTLKQIVIKLREQF-ENKTIVFNHSSGGDPEIVMHSFNCGGEFFYCNSTQLFNSTWNNNT---EGSNNTEG-NTITLPCRIKQI
LAI    :  AKWNATLKQIASKLREQFGNNKTIIFKQSSGGDPEIVTHSFNCGGEFFYCNSTQLFNSTWFNSTWSTEGSNNTEGSDTITLPCRIKQF
KNH1144:  SQWNKTLQQVAEQLREHF-KNKTIIFNSSSGGDLEITTHSFNCGGEFFYCNTSGLFNSTW-NTSMS--GSSNTETNGTITLQCRIKQI

                            450                                                  500
JR-FL  :  INMWQEVGKAMYAPPIRGQIRCSSNITGLLLTRDGGINEN-GTEIFRPGGGDMRDNWRSELYKYKVVKIEPLGVAPTKAKRRVVQREKR
LAI    :  INMWQEVGKAMYAPPISGQIRCSSNITGLLLTRDGGNN-NNGSEIFRPGGGDMRDNWRSELYKYKVVKIEPLGVAPTKAKRRVVQREKR
KNH1144:  INMWQRTGQAIYAPPIQGVIRCESNITGLLLTRDGGEEKNSTNEIFRPGGGDMRDNWRSELYKYKVVKIEPLGVAPTRARRRVVGREKR
```
